# The cytoprotective protein MANF promotes neuronal survival independently from its role as a GRP78 cofactor

**DOI:** 10.1016/j.jbc.2021.100295

**Published:** 2021-01-15

**Authors:** Ave Eesmaa, Li-Ying Yu, Helka Göös, Kristofer Nõges, Vera Kovaleva, Maarit Hellman, Richard Zimmermann, Martin Jung, Perttu Permi, Markku Varjosalo, Päivi Lindholm, Mart Saarma

**Affiliations:** 1 Institute of Biotechnology, HiLIFE, University of Helsinki, Helsinki, Finland; 2 Department of Chemistry, Nanoscience Center, University of Jyväskylä, Jyväskylä, Finland; 3 Medical Biochemistry and Molecular Biology, Saarland University, Homburg, Germany; 4 Department of Biological and Environmental Science, Nanoscience Center, University of Jyväskylä, Jyväskylä, Finland

**Keywords:** mesencephalic astrocyte-derived neurotrophic factor, GRP78, unfolded protein response, endoplasmic reticulum stress, dopamine neurons, neuronal cell death, protein–protein interaction, neuroprotection, apoptosis, ATP, AP-MS, affinity purification mass spectrometry, BiFC, bimolecular fluorescence complementation, C-MANF, C-terminal domain of MANF, CSPs, chemical shift perturbations, DA, dopamine, ER, endoplasmic reticulum, GDNF, glial cell line–derived neurotrophic factor, GFP-SH, SH-tagged GFP, IRE1, inositol-requiring enzyme 1, MANF, mesencephalic astrocyte-derived neurotrophic factor, MST, microscale thermophoresis, NBD, nucleotide-binding domain, NEI, nucleotide exchange inhibitor, NMR, nuclear magnetic resonance, NPTN, neuroplastin, PDIA1, protein disulfide isomerase family A member 1, PDIA6, protein disulfide isomerase family A member 6, PERK, protein kinase RNA-like ER kinase, PPIs, protein-protein interactions, SBD, substrate-binding domain, SCG, superior cervical ganglion, Tg, thapsigargin, TH, tyrosine hydroxylase, Tm, tunicamycin, UPR, unfolded protein response

## Abstract

Mesencephalic astrocyte-derived neurotrophic factor (MANF) is an endoplasmic reticulum (ER)-stress–regulated protein exhibiting cytoprotective properties through a poorly understood mechanism in various *in vitro* and *in vivo* models of neuronal and non-neuronal damage. Although initially characterized as a secreted neurotrophic factor for midbrain dopamine neurons, MANF has recently gained more interest for its intracellular role in regulating the ER homeostasis, including serving as a cofactor of the chaperone glucose-regulated protein 78 (GRP78). We aimed for a better understanding of the neuroprotective mechanisms of MANF. Here we show for the first time that MANF promotes the survival of ER-stressed neurons *in vitro* as a general unfolded protein response (UPR) regulator, affecting several UPR pathways simultaneously. Interestingly, MANF does not affect naïve neurons. We hypothesize that MANF regulates UPR signaling toward a mode more compatible with neuronal survival. Screening of MANF interacting proteins from two mammalian cell lines revealed a conserved interactome of 15 proteins including several ER chaperones such as GRP78, GRP170, protein disulfide isomerase family A member 1, and protein disulfide isomerase family A member 6. Further characterization confirmed previously published finding that MANF is a cofactor of GRP78 interacting with its nucleotide binding domain. Using microscale thermophoresis and nuclear magnetic resonance spectroscopy, we discovered that MANF is an ATP binding protein and that ATP blocks the MANF–GRP78 interaction. Interestingly, functional analysis of the antiapoptotic properties of MANF mutants in cultured neurons revealed divergent roles of MANF as a GRP78 cofactor and as an antiapoptotic regulator of UPR. We conclude that the co-factor type interaction with GRP78 is dispensable for the survival-promoting activity of MANF in neurons.

Mesencephalic astrocyte-derived neurotrophic factor (MANF, also known as arginine-rich, mutated in early stage tumors—ARMET) was originally characterized as a protein secreted from the rat type-1 astrocyte ventral mesencephalic cell line as a growth factor able to promote the survival of cultured midbrain dopamine (DA) neurons (1). To date, MANF has been shown to be cytoprotective in several neuronal and non-neuronal disease models such as Parkinson’s disease, spinocerebellar ataxia, ischemic stroke, diabetes, myocardial infarction, and retinal degeneration (2, 3, 4, 5, 6, 7, 8)

MANF is well conserved in evolution both in invertebrate and vertebrate species (1, 9). This is illustrated by the finding that human MANF overexpression was able to rescue larval lethality resulting from *Drosophila Manf* deletion in a fruit fly (10). MANF has an amino-terminal (N-terminal) signal peptide that is cleaved after having directed the protein to the endoplasmic reticulum (ER), giving rise to mature protein of about 18 kDa (1, 11, 12). As a characteristic feature, MANF has eight cysteine residues with conserved spacing, forming four disulfide bridges (11, 13, 14). Structurally, MANF has two domains—the N-terminal domain being a structural homolog to saposin-like proteins, whereas the closest structural homolog to its carboxy-terminal (C-terminal) domain are proteins belonging to the SAF-A/F, Acinus, and PIAS (SAP) protein superfamily (14, 15, 16, 17). Additionally, MANF features a KDEL-like ER retention sequence at its very C terminus and a flexible linker region connecting the two domains (15, 17, 18).

siRNA-mediated silencing of MANF rendered cultured cells more vulnerable to ER stress–induced death (19). ER stress is a collective term for disturbances in the ER homeostasis that can be brought on by a variety of cellular insults such as ER calcium disbalance, aberrations in ER protein glycosylation, abnormal protein expression or folding, virus infections, and pharmacological agents. Cells respond to ER stress by activating signaling cascades known as the unfolded protein response (UPR) (reviewed in (20)). In mammalian cells, UPR signaling occurs through three ER transmembrane receptors—inositol-requiring enzyme 1 (IRE1), protein kinase RNA-like ER kinase (PERK), and activating transcription factor 6 (ATF6) (reviewed in (21)). UPR is an adaptive response aiming first to restore the ER homeostasis by reducing the misfolded protein load and increasing the folding capacity, but if stress is persistent, ER stress-induced apoptosis is launched (reviewed in (22)). Glucose-regulated protein 78 (GRP78, also known as BiP; gene *HSPA5*), a major ER chaperone, interacts with three UPR sensors and regulates their activation. Under normal conditions, GRP78 has been shown to keep IRE1 and PERK inactive by binding to their ER lumenal domains, thus repressing their dimerization and subsequent autophosphorylation (23, 24). Accordingly, under conditions of increased misfolded protein load, GRP78 is lured away from IRE1 and PERK lumenal domains by preferential binding to misfolded proteins, leading to activation of IRE1 and PERK pathways (23). Additionally, making the mechanism of UPR activation more complex, a growing number of evidence suggests that IRE1 can be activated by directly interacting with unfolded proteins (25, 26). It has been proposed that GRP78 keeps ATF6 inactive by binding to and masking the Golgi-localization signal of ATF6 (27, 28). The dissociation of GRP78 from ATF6 leads to the translocation of ATF6 to Golgi, where it gets processed, subsequently moves to the nucleus and acts as a transcription factor (29, 30).

GRP78 is a member of the heat shock protein 70 kDa (Hsp70) family and consists of two major, functionally distinct domains. The N-terminal nucleotide-binding domain (NBD) contains an ATP catalytic site responsible for the ATPase activity of GRP78. The C-terminal domain consists of the substrate binding pocket and a lid forming the substrate-binding domain (SBD) (31). In its ATP-bound state, GRP78 has a low affinity for substrate polypeptides. ATP hydrolysis to ADP increases the affinity, allowing for subsequent binding and folding of substrates. ADP exchange to ATP, catalyzed by the nucleotide exchange factors, completes the cycle and releases the substrate protein (32, 33). The ATPase cycle and thus the chaperone activity of GRP78 is controlled by its interactions with different co-factors. The most common co-factors of GRP78 are Hsp40 family co-chaperones and the nucleotide exchange factors GRP170 (glucose-regulated protein 170 kDa, also known as HYOU1 and ORP150) and SIL1 (reviewed in (34)).

ER-stress has been shown to induce the mRNA and protein levels of MANF in several cell and tissue types both *in vitro* and *in vivo* (4, 12, 13, 19, 35, 36, 37). A role of MANF in the regulation of UPR signaling is further supported by the activation of UPR observed in MANF-deficient mice and fruit flies (38, 39, 40). Both conventional *Manf*^−/−^ and pancreas-specific *Pdx-1 Cre*::*Manf*^*fl/fl*^ mice develop type I diabetes because of progressive postnatal loss of pancreatic beta cell mass. Chronic, unresolved ER stress has been proposed to be a major cause of beta cell loss as pancreatic islets of *Manf*^−/−^ mice show activation of UPR genes. Additionally, in a recently published study, MANF was shown to reduce the cytokine-induced ER stress and death of human pancreatic islets (41).

Although increasingly studied over the past 15 years, the molecular mechanism of MANF cell survival–promoting activity remains poorly understood. Mapping of the protein–protein interactions (PPIs) is commonly used to understand the cellular functions of proteins. To date, very little is known about the MANF PPIs and their biological functions. We have recently shown neuroplastin (NPTN) to be a plasma membrane receptor for MANF (42), and another study suggested that the binding of MANF to the plasma membrane is modulated by KDEL endoplasmic reticulum protein retention receptors (43). In cellular models of multiple epiphyseal dysplasia MANF interacts with a V194D mutant of matrilin-3 that forms non-native disulfide bonds, suggesting that MANF functions as a part of ER stress response with other chaperones (36).

Using chemical cross-linking and immunoprecipitation, MANF was shown to interact with the major ER chaperone GRP78 (4). It was proposed that the interaction with GRP78 is calcium-dependently regulating MANF secretion from the ER. A recent study identified MANF as the nucleotide exchange inhibitor (NEI) of GRP78 that functions by stabilizing the ADP-bound or apo-conformations of the chaperone, possibly prolonging the folding time-window for some substrate polypeptides, thus helping to maintain protein homeostasis (44).

To understand how MANF supports cell survival, we screened for and characterized MANF PPIs using affinity purification mass spectrometry (AP-MS) (45, 46) in two mammalian cell lines, bimolecular fluorescence complementation (BiFC) assay and microscale thermophoresis (MST). In addition to interacting with GRP78 and GRP170, MANF also interacts with other ER-resident chaperones such as protein disulfide isomerase family A member 6 (PDIA6) and protein disulfide isomerase family A member 1 (PDIA1). We investigated the GRP78 cofactor role of MANF and verified that MANF is a cofactor of GRP78. We also demonstrate for the first time that MANF promotes the survival of cultured DA and superior cervical ganglion (SCG) neurons by regulating UPR pathways. Using MST and nuclear magnetic resonance (NMR) spectroscopy, we show that MANF is an ATP binding protein and ATP blocks MANF interaction with GRP78. We suggest that the ATP-binding properties of MANF warrant further studies as these might have potential implications to its biological function. To our surprise, mutating the amino acid residues R133 and E153, shown to be critical for GRP78-binding (44), did not abolish the survival-promoting activity of MANF in tunicamycin (Tm)-treated SCG neurons. This indicates that MANF has an additional mechanism, unrelated to its interaction with GRP78, for rescuing neurons from ER-stress triggered apoptosis. We thus propose that although MANF acts as a cofactor of GRP78, it exerts its survival-promoting function by regulating UPR signaling.

## Results

### Activation of PERK and IRE1 mediate MANF neuroprotective effect against tunicamycin-induced ER stress in cultured sympathetic neurons

**Figure 1:**
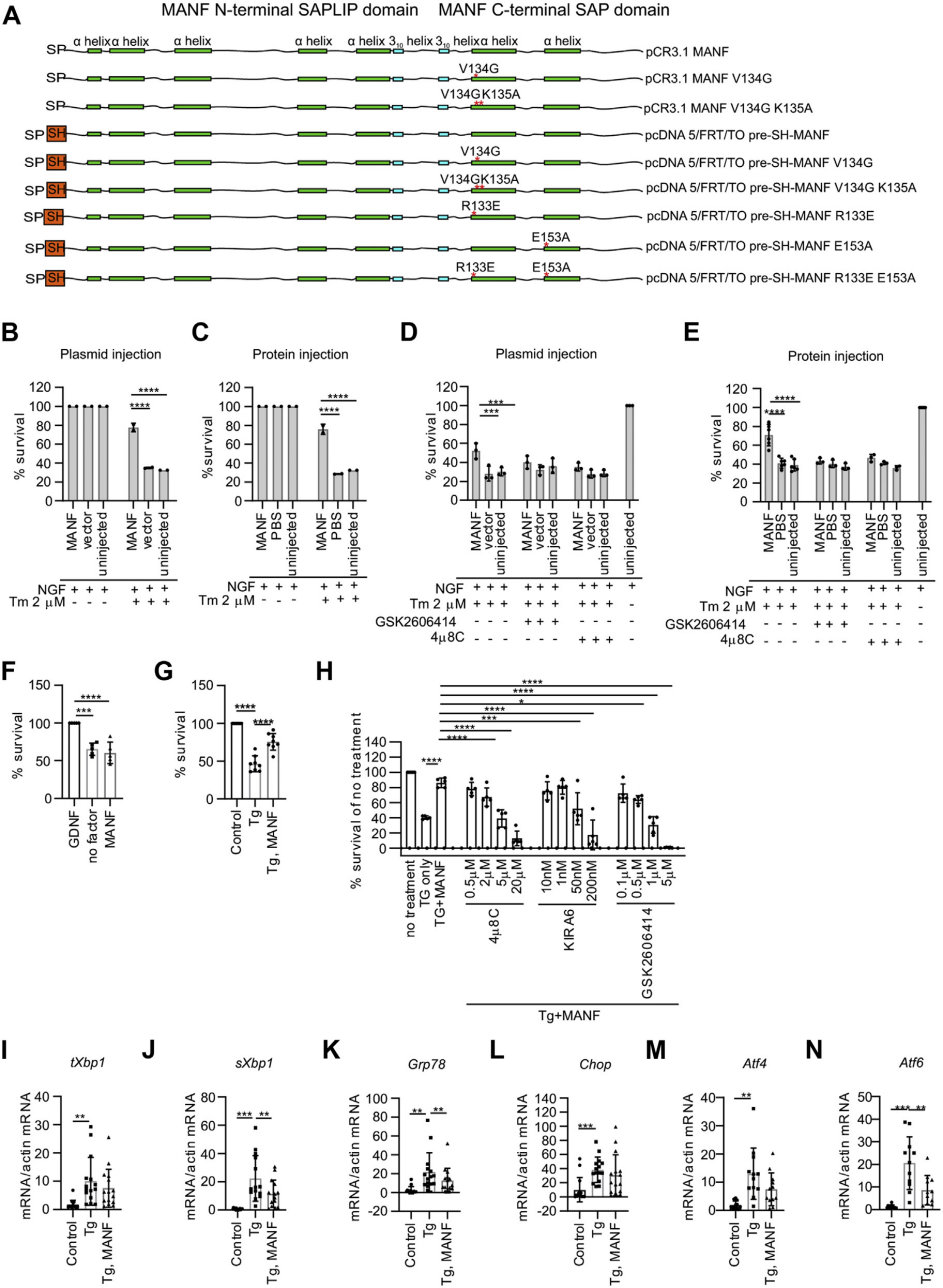
**MANF is an UPR-regulating protein in primary neuron cultures.***A*, a schematic depiction of MANF expression constructs used in this study with *red asterisks* indicating a point mutation. The domains of MANF are shown above the pCR3.1 MANF construct scheme. Shown are also ER signal peptide (SP), the Twin-StrepII-HA tag (SH) in pre-SH-MANF constructs, and secondary structure elements: α (*green bars*)- and 3_10_ (*blue bars*) helices. *B*, mouse SCG neurons maintained in the presence of NGF were treated with tunicamycin and microinjected with (*B*) MANF expression plasmid or (*C*) recombinant MANF protein. Mouse SCG neurons maintained in the presence of NGF were microinjected with (*D*) the indicated expression plasmids or (*E*) recombinant human MANF protein, and treated with 2 μM tunicamycin and 2 μM PERK signaling inhibitor GSK2606414 or 25 μM IRE1 signaling inhibitor 4μ8C. The number of living injected, fluorescent neurons was calculated 72 h after the injections and expressed as the percentage of initially injected neurons. Shown are the means of two to six experiments ± SD MANF plasmid or protein injected groups were compared with the empty vector or PBS injected controls of the same treatment group using one-way analysis of variance (ANOVA) and Sidak’s multiple comparison *post hoc* test. ∗∗∗ denotes *p* < 0.001, ∗∗∗∗ denotes *p* < 0.0001. The null hypothesis was rejected at *p* < 0.05. *F*, E13 midbrain floor neuron cultures were cultured with MANF (100 ng/ml), GDNF (100 ng/ml), or no neurotrophic compound (no factor) for 5 days. Dopamine (DA) neurons were identified by tyrosine hydroxylase (TH)-immunostaining and expressed as % of cell survival in each condition compared with the positive control, GDNF-treated neurons. Shown are the means ± S.D. of five independent experiments per condition. ANOVA and Tukey’s multiple comparison post hoc test. *G*, MANF protein protects embryonic dopamine neurons from ER stress. Dissociated cultures of E13.5 NMRI mouse midbrain floors were grown for 5 days and then treated with 100 nM thapsigargin (Tg) for 3 days. After 3 days, the cultures were immunostained for TH. TH-positive neurons were counted and expressed as a percentage of nontreated neurons. Shown are the means of eight experiments ± SD. Tg-treated group was compared with control group and Tg, MANF group using ANOVA and Dunnett’s multiple comparison *post ho*c test. *H*, E13.5 DA neurons were cultured without any trophic factors for 5 days, then treated 3 days as indicated with Tg, MANF, IRE1 (4μ8C or KIRA6), or PERK (GSK2606414) inhibitors. The results are expressed as percentage of TH-positive cell survival as compared with the non-Tg treated condition. Data of each treatment groups were compared with Tg+MANF group, n = 5, ordinary one-way ANOVA and Sidak’s multiple comparisons *post hoc* test. *I*–*N*, DA neurons were cultured 5 to 7 days *in vitro*, then ER stress was induced by adding 200 nM thapsigargin (Tg). MANF was added to the cultures at the same time as Tg. RNA was isolated after 24 h. The expression levels of ER stress marker transcripts were normalized to levels of β-actin in the same samples. Shown are means of n = 11 to 15 experiments ±SD. ANOVA and Tukey’s multiple comparison *post hoc* test. ∗, ∗∗, ∗∗∗, ∗∗∗∗ denote *p* < 0.05, *p* < 0.01, *p* < 0.001, *p* < 0.0001, respectively. ER, endoplasmic reticulum; GDNF, glial cell line–derived neurotrophic factor; IRE1, inositol-requiring enzyme 1; MANF, mesencephalic astrocyte-derived neurotrophic factor; NGF, nerve growth factor; PERK, protein kinase RNA-like ER kinase; SCG, superior cervical ganglion; UPR, unfolded protein response.

Overexpression of MANF by plasmid or protein microinjection into SCG neurons has been shown to promote their survival against serum deprivation, topoisomerase II inhibitor etoposide, and protein kinase inhibitor staurosporine, whereas MANF added to the culture medium has no effect on the survival of SCG neurons (15, 47). Despite MANF being an ER-stress regulated protein, the effect of MANF against ER stress-induced death in SCG neurons has not been reported. Here, we investigated the neuroprotective effects and mechanisms of MANF in SCG neurons in an ER stress-related apoptosis paradigm. Neurons were treated with Tm, which is an inhibitor of N-linked glycosylation, causing accumulation of misfolded glycoproteins in the ER lumen and eventually apoptosis through activation of UPR (for a review see (48)). First, we tested the effect of MANF plasmid and then protein microinjection to neuron survival without Tm treatment. MANF microinjection did not affect neuronal survival as compared with naïve or vector injected neurons (Fig. 1, *B* and *C*). As expected, Tm-treatment decreased the survival of SCG neurons to ∼30% compared with untreated neurons. The survival of Tm-treated SCG neurons injected with MANF plasmid (Fig. 1, *A* and *B*) or MANF protein (Fig. 1*C*) was significantly increased as compared with neurons injected with pCR3.1 control plasmid or PBS, respectively. Thus, while MANF had no effect on the survival of naïve neuronal cultures, it efficiently rescued Tm-treated neurons from apoptosis, regardless of whether it was injected as a plasmid or as a recombinant protein (Fig. 1, *B* and *C*).

MANF has been mostly studied for its neuroprotective properties or as an UPR-regulated ER-resident protein, but the mechanistic link between those functions has remained elusive. We hypothesized that the neuroprotective effect of MANF may arise from its ability to cross-talk with the UPR machinery. Therefore, to investigate the mechanism of the survival-promoting effect of MANF, we tested whether it is dependent on the activity of PERK- and IRE1-mediated UPR signaling pathways. For this, UPR signaling was dampened by adding either GSK2606414, an inhibitor of PERK signaling (49), or 4μ8C, an inhibitor of IRE1 signaling (50). The protective effect of MANF against Tm was lost on addition of either of the inhibitors, indicating that the activity of both PERK and IRE1 pathways are necessary for the survival-promoting activity of MANF in SCG neurons against ER stress (Fig. 1*D*). Similarly, inhibiting either PERK signaling or IRE1 signaling abolished the protective effect of recombinant MANF protein (Fig. 1*E*). We therefore conclude that the intracellular neuroprotective activity of MANF in ER-stressed SCG neurons is dependent on its ability to cross-talk with the PERK and IRE1 pathways of UPR signaling. These data thus comprise the first line of evidence that the survival-promoting mechanism of MANF relies on the UPR signaling.

### Extracellularly added MANF promotes the survival of dopamine neurons and decreases expression of UPR genes in thapsigargin-induced ER stress

Intracellularly, MANF localizes to the ER lumen (19, 47). However, most studies investigating the cytoprotective function of MANF have employed an extracellular application mode of MANF. For internalization, MANF has been suggested to rely on sulfolipids and plasma membrane KDEL receptors (43, 51). Nevertheless, it has remained unclear whether the intracellularly and extracellularly applied MANF rely of the same intracellular counterparts to elicit survival-promoting effect. Therefore, to investigate the effect of extracellularly added MANF on neuronal survival during ER stress, we used mouse embryonic midbrain DA neuronal cultures, shown to respond to MANF (1). First, we tested the survival-promoting effect of MANF on naïve DA cultures after serum deprivation. Glial cell line–derived neurotrophic factor (GDNF) has been shown to promote the survival of midbrain DA cultures and was used as a positive control (52). Unlike GDNF, MANF did not increase the survival of naïve DA neurons in culture (Fig. 1*F*). Next, ER stress and UPR activation were induced by adding thapsigargin (Tg) to the culture media. Tg is a selective inhibitor of the SERCA (sarco/ER Ca^2+^ ATPase) inducing ER Ca^2+^ disbalance and subsequently apoptosis (53). Tg-treatment reduced the survival of DA neurons by more than 50%, whereas recombinant MANF protein added to the culture media of Tg-treated DA neurons significantly promoted neuron survival (Fig. 1*G*). Thus, we show for the first time that recombinant MANF protein is able to rescue DA neurons against ER stress-induced death *in vitro*. What is more, our data show that MANF has no survival-promoting effect on naïve DA neurons *in vitro*, indicating that neuronal ER stress is needed for MANF to be able to exert its antiapoptotic properties.

Next, to study whether, similar to intracellularly delivered MANF, extracellularly applied MANF relies on UPR signaling, we used inhibitors of IRE1 and PERK pathways. Different concentrations of 4μ8C and KIRA6 were used to inhibit the RNase and kinase activities of IRE1, respectively (50, 54), and GSK2606414 was used to inhibit PERK signaling as previously. Additionally, the DA neuron cultures were treated with Tg and MANF. Like with SCG neurons, MANF was able to rescue DA neurons from ER stress–induced apoptosis, but the protective effect of MANF was lost upon presence of any of the tested UPR inhibitors, especially at higher concentrations (Fig. 1*H*). These findings indicate that regardless of the application mode, the neuroprotective activity of MANF against ER-stress induced apoptosis relies on its ability to activate survival-promoting signaling through UPR pathways.

To further elucidate the mechanism how extracellularly applied MANF is able to rescue ER-stressed DA neurons, we performed qPCR analysis of transcripts corresponding to three pathways of UPR signaling. The ribonuclease activity of IRE1 directly splices *Xbp1* mRNA, resulting in transcriptionally active spliced *Xbp1* (s*Xbp1*), which in turn regulates the expression of IRE1 downstream genes such as *Grp78*. *Atf4* and *Chop* are transcription factors corresponding to activation of PERK pathway, whereas changing levels of *Atf6* reflect changes in the ATF6 pathway activation (48). Correspondingly, Tg treatment significantly increased the mRNA levels of total *Xbp1* (t*Xbp1*) and spliced *Xbp1*, *Chop*, *Grp78*, *Atf4*, and *Atf6* in the DA cultures (Fig. 1, I–*N*), whereas exogenously added MANF was able to reduce the mRNA levels of spliced *Xbp1*, *Grp78*, and *Atf6* in Tg-treated cultures (Fig. 1, *J*, *K* and *N*). Interestingly, while the change in *Chop* and *Atf4* transcripts did not reach statistical difference, we observed reduction of these PERK pathway–regulated mRNAs in cultures treated with MANF and Tg when compared with Tg-treated DA cultures. It is, therefore, noteworthy, that MANF can regulate several UPR pathways, especially IRE1 and ATF6, simultaneously. These data also indicate that at least in cultured DA neurons, the mechanism of action of MANF is not dependent on its mode of application as both intracellulary and extracellularly applied MANF relies on the intactness of UPR signaling.

### Generation of HEK293 and INS1 cell lines for inducible overexpression of MANF for AP-MS

**Figure 2:**
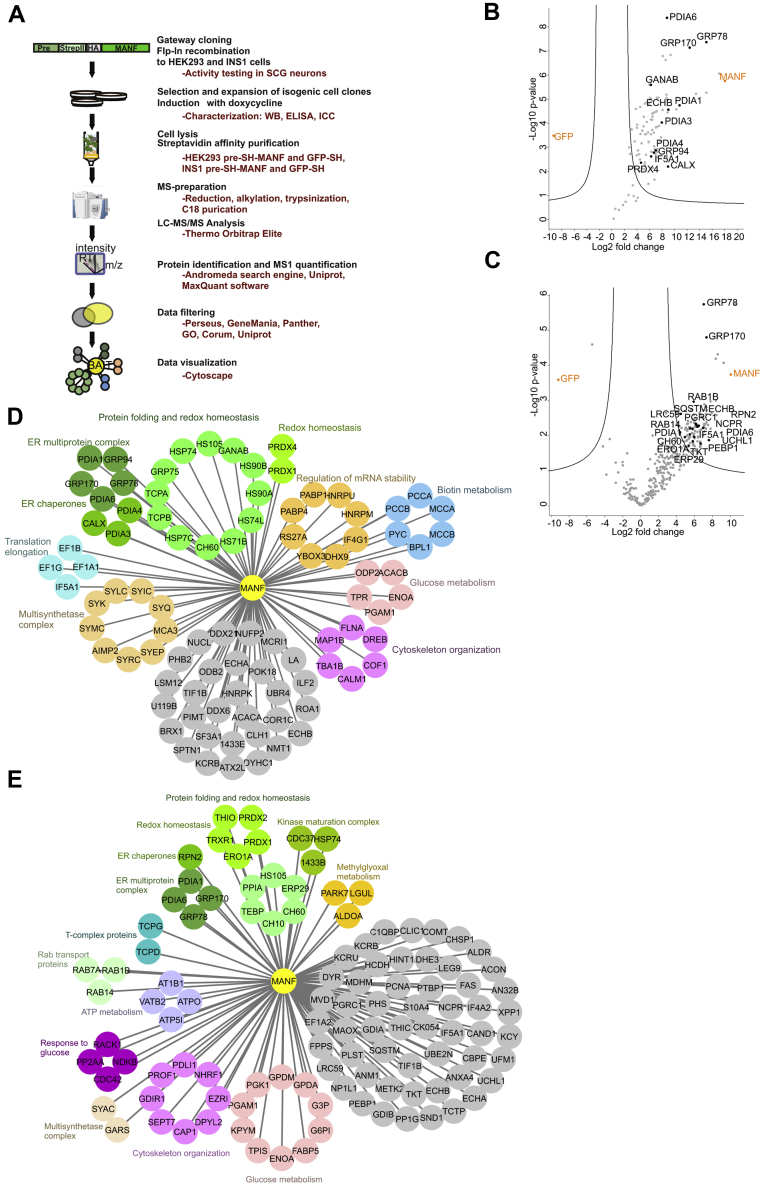
**Interaction proteome of MANF in HEK293 and INS1 pre-SH-MANF cells.***A*, schematic pipeline of the AP-MS. Signal peptide (PRE), StrepII, and HA tag are indicated. *B*–*C*, volcano plots showing the protein abundance (log2 fold change) against the *t*-statistic significance (−log10 *p*-value) after AP-MS analysis in (*B*) HEK293 and (*C*) INS1 cells. AP-MS from the respective GFP-SH cell lines served as a negative control. Significantly enriched proteins forming the MANF interactome were calculated using the Volcano plot plugin of Perseus software package (FDR ≤ 0.001, S0 = 2) and are shown on the right side of the respective parabola. Bait names (MANF and GFP) are shown in *orange*. The UniProt entry names of the ER-localized or ER-associated proteins of the MANF interactomes are shown. Interaction networks of MANF in (*D*) HEK293 or (*E*) INS1 cells. Nodes representing functionally proteins as determined by CORUM complex database, existing literature and statistically significantly overrepresented GO term analysis are grouped together, indicated with a similar color and a group name. CORUM database of mammalian protein complexes was used to identify nodes representing subunits of protein complexes enriched in the MANF interactomes. Complexes are indicated by a complex name. AP-MS, affinity purification mass spectrometry; ER, endoplasmic reticulum; FDR, false discovery rate; GFP-SH, SH-tagged GFP; MANF, mesencephalic astrocyte-derived neurotrophic factor.

To provide more insight into the MANF mechanism of action, we aimed to characterize its PPIs in human embryonic kidney HEK293 and rat insulinoma INS1 cell lines using AP-MS. The workflow of AP-MS is presented in (Fig. 2*A*). To facilitate affinity purification, we inserted an SH-tag comprising of Twin-StrepII-tag (IBA GmbH) followed by a hemagglutinin tag between the sequences coding for signal peptide (pre) and mature regions of human MANF, respectively (Figs. 1*A* and 2A).

We used the SH-tagged MANF construct to generate stable isogenic doxycycline-inducible cell lines for AP-MS (Fig. 2*A*). Flp-In T-REx HEK293 (HEK293 parental, Invitrogen) and Flp-In T-REx INS1 #5-3.19 (INS1 parental (55)) were used as parental cell lines as both contain a single genomic FRT site for Flp-recombinase–mediated targeted genomic insertion of a gene of interest. For AP-MS, HEK293 parental cell line is a commonly used and easily available cell line (45, 46). INS1 was chosen because of the previously published data showing the crucial importance of MANF in the development and maintenance of mouse pancreatic insulin producing β-cells (39). Cell lines expressing SH-tagged MANF and originating from HEK293 or INS1 parental cell lines were named HEK293 pre-SH-MANF and INS1 pre-SH-MANF, respectively. HEK293 and INS1 cell lines expressing SH-tagged GFP (GFP-SH) were generated and used as a control. Altogether 4 doxycycline-inducible cell lines were used for AP-MS: (1) HEK293 pre-SH-MANF, (2) HEK293 GFP-SH, (3) INS1 pre-SH-MANF, and (4) INS1 GFP-SH.

### MANF interactomes of 90 and 109 proteins identified from HEK293 and INS1 cells, respectively

To characterize the interactome of MANF, we used the SH-tagged MANF and GFP as baits in our AP-MS experiments. From the HEK293 pre-SH-MANF and HEK293 GFP-SH cell lines, a total of 214 proteins were identified and quantified (Table S1). Initial filtering by removal of keratins, ribosomal proteins, and other common contaminants further reduced the list to 109 proteins. Next, to discriminate between the background and significantly enriched proteins, we plotted the base2 logarithmized fold change ratios of each protein in pre-SH-MANF/SH-GFP cell lines against their respective −log10 (*p*-values). To correct for multiple testing, a permutation-based false discovery rate was applied with the following parameters: false discovery rate (FDR) ≤0.001, s0 = 2. Significant hits from HEK293 cell line are shown on the right side of the volcano plot parabola. In total, the MANF high-confidence interactome in HEK293 cells comprised of 90 proteins (Fig. 2*B*, Table S2). Expectedly, MANF as the bait protein was the most enriched proteins in pull-down data set from the HEK293 cell line. Analysis of subcellular localization data associated with each protein in the UniProtKB database revealed a total of 12 ER-localized or ER-associated proteins among the interacting proteins. All three members of the human ER-resident glucose regulated protein family—the GRP78, GRP170, and GRP94—were present, with the first two being the most enriched proteins in the HEK293 MANF interactome. Additionally, the ER-resident protein disulfide isomerases were represented by PDIA1, protein disulfide isomerase family A member 3, protein disulfide isomerase family A member 4, and PDIA6. Other than these, the other five ER-resident or ER-associated proteins in the HEK293 MANF interactome were trifunctional enzyme subunit beta (ECHB), neutral alpha-glucosidase AB, peroxiredoxin-4, calnexin, and eukaryotic translation initiation factor 5A-1 (IF5A1) (Fig. 2*B*).

From INS1 cell lines, a total of 567 proteins were identified and quantified when SH-MANF and SH-GFP were used as baits for pull-down experiments (Table S3). Initial filtering and volcano plot were constructed following the same principles as with HEK293 cell line. Significant hits, forming the high-confidence interactome of MANF in INS1 cells—a total of 109 proteins—are shown on the right side of the volcano plot parabola (Fig. 2*C* and Table S4).The MANF interactome in INS1 cells contained a total of 19 ER-resident or ER-associated proteins. As in HEK293 cells, GRP78 and GRP170 were the most enriched proteins in the INS1 MANF interactome. In addition, ECHB, PDIA6, PDIA1, and IF5A1 were the ER-resident or ER-associated proteins present in INS1 MANF interactome as well as in the HEK293 MANF interactome. In addition to these, the other 12 ER-resident or ER-associated proteins in the INS1 interactome were ribophorin 2, ras-related protein Rab-1B, ubiquitin carboxyl-terminal hydrolase isozyme L1, membrane-associated progesterone receptor component 1, sequestosome-1, ras-related protein Rab-14, leucine-rich repeat-containing protein 59, NADPH--cytochrome P450 reductase, phosphatidylethanolamine-binding protein 1, 60 kDa heat shock protein (CH60), transketolase, ERO1-like protein alpha, and endoplasmic reticulum resident protein 29 (Fig. 2*C*). Overall, while both MANF interactomes contained more than 10% ER-localized or ER-associated proteins, including a number of new MANF interactors, ER chaperones GRP78 and GRP170 stand out as the most enriched proteins in both pull-down data sets. Both GRP78 and GRP170 are UPR-regulated chaperones and have been reported before as interactors of MANF (36, 44). These findings are, thus, well in agreement with previously published data about MANF interacting with ER chaperones.

### MANF-interacting proteins are involved in protein folding processes

Next, the high-confidence interactors of MANF were functionally grouped based on statistically significantly overrepresented GO terms, CORUM protein complex database, and existing literature (Fig. 2, *D* and *E*). Functional grouping indicted that in both HEK293 and INS1 cells MANF-interacting proteins are involved in a variety of cellular processes. Notably, proteins performing protein folding and redox homeostasis processes were well represented in both interactomes, as were proteins involved in glucose metabolism and cytoskeleton organization. These data suggest that MANF, too, might be involved in cellular metabolism and protein folding processes-a notion well compatible with the phenotype of MANF-deficient mice (39).

### Conserved interactome of MANF containing 15 proteins common in HEK293 and INS1 cells

**Figure 3:**
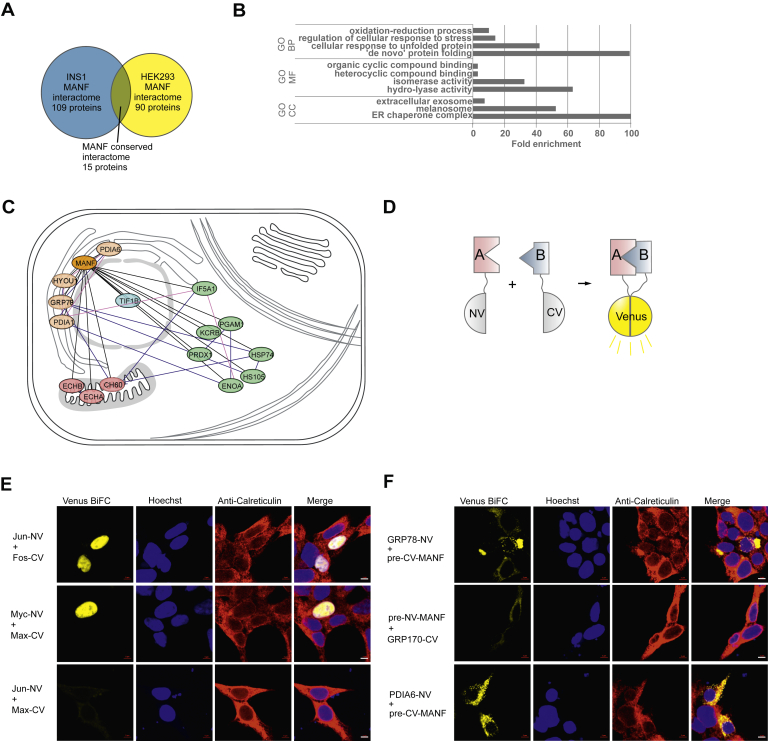
**The conserved interactome of MANF.***A*, Venn diagram illustrating the number of proteins interacting with MANF in HEK293 and INS1 cells. The conserved interactome is formed of 15 proteins. *B*, GO term overrepresentation analysis of the MANF conserved interactome. Significantly overrepresented (Bonferroni corrected *p*-value < 0.05) terms are shown with GO terms with fold enrichment values of ≥100 presented as 100. *C*, cellular compartment layout mapping and interaction network of the MANF conserved interactome. Interactions identified in this study are indicated with *black lines* between nodes. The network was supplemented with node–node physical interaction (*blue lines*) and colocalization (*pink lines*) data provided by analysis with Genemania (www.genemania.com) online platform. UniProtKB (ww.uniprot.org) database and published literature were used to assign proteins to cellular compartments. Nodes in different cellular compartments are color-coded as follows: ER-*orange*; mitochondrial-*pink*; cytoplasmic-*green*. *D*, the principle of signal formation in the BiFC assay. Putative interaction partners (proteins A and B) are tagged with either the N-terminal (NV) or C-terminal (CV) fragment of Venus fluorescent protein. Venus signal is reconstituted when proteins are in close proximity, indicating either direct interaction or being in the same protein complex at permissive proximity. *E*, positive (Jun-NV and Fos-CV; Myc-NV and Max-CV) and negative (Jun-NV and Max-CV) controls of BiFC signal (*yellow*) formation. *F*, MANF gave a positive BiFC signal with GRP78, GRP170, and PDIA6. Nuclei were visualized by Hoechst 33342 staining (*blue*), and the ER by anticalreticulin immunofluorescence staining (*red*). Scale bars denote 10 μm. BiFC, bimolecular fluorescence complementation; ER, endoplasmic reticulum; GRP, glucose-regulated protein; MANF, mesencephalic astrocyte-derived neurotrophic factor.

We identified 90 and 109 proteins from HEK293 and INS1 cells, respectively, as the putative interacting proteins for MANF. In an effort to narrow down the list of MANF-interacting proteins for further analysis, we looked at the intersection of a Venn diagram consisting of the MANF interactomes in the 2 cell lines studied. A total of 15 proteins were interacting with MANF in both HEK293 and INS1 cell lines (Fig. 3*A*). This subset of interacting proteins we termed the conserved interactome of MANF.

Analysis of statistically significantly (*p* < 0.05) overrepresented GO terms in the conserved interactome of MANF revealed the ER chaperone complex as the most overrepresented cellular compartment GO term (56). Correspondingly, *de novo*’ protein folding, cellular response to unfolded protein, and regulation of cellular response to stress were the most overrepresented biological process GO terms (Fig. 3*B*).

The MANF conserved interaction network was supplemented with intranetwork node–node co-localization and physical interaction data using the Genemania website (57). Combining protein localization data in UniprotKB database and, if needed, existing literature, we mapped the individual proteins forming the MANF conserved interactome to their respective subcellular compartments (Fig. 3*C*). Intracellularly, MANF has been shown to localize to the ER lumen and is consequently more likely to interact with proteins similarly localized to the ER, either transiently or as *bona fide* ER proteins. Additionally, data presented here and previously suggest ER being the *locus operandi* for the cytoprotective activity of MANF (12, 19, 35, 36, 37, 39). Therefore, we focused on ER-localized fraction of MANF conserved interactome for subsequent analysis. Mapping the MANF conserved interactome proteins to their subcellular locations revealed four proteins, in addition to MANF, with ER lumenal localization. Those proteins were GRP78, GRP170, PDIA1, and PDIA6. GRP78, GRP170, and PDIA6 have previously been reported to interact with MANF, albeit information is still lacking regarding the cellular context of these interactions (4, 36, 44).

### MANF interacts with GRP78, GRP170, and PDIA6 in the ER as shown by BiFC

To further validate the MANF conserved interactome, we used the BiFC assay in HEK293 cells. For this, we used a split Venus-based BiFC approach where the bait and prey proteins were fused with either the N-terminal (NV) or C-terminal (CV) fragment of the Venus fluorescent protein (Fig. 3*D*). To enable correct subcellular localization of fusion proteins, we took into account existing data in the UniProt database about protein processing and topology. Therefore, proteins with known ER-targeting signal peptide or with mitochondrial targeting sequence in their N termini were C terminally fused with the respective fragments of Venus fluorescent protein. MANF-coding constructs had the Venus fragment inserted between the signal peptide and the mature MANF protein to allow for co-translational removal of the signal peptide, resulting in mature MANF N terminally tagged with a Venus fragment. Out of 15 proteins forming the conserved interactome of MANF, we tested 11 by BiFC.

As a positive control for BiFC signal formation in cells, we used the Jun and Myc pair as well as the Myc and Max pair of transcription factors interactions of which have been well studied with BiFC among other methods (58, 59). The nonspecific background for BiFC signal was visualized by using a pair of noninteracting Jun and Max transcription factors (Fig. 3*E*).

GRP78, GRP170, and PDIA6 have a manually annotated ER localization (www.uniprot.org) and all gave a positive BiFC signal that colocalized with anti-Calreticulin immunostaining (Fig. 3*F*). With three of the tested proteins, we observed a positive BiFC signal that did not co-localize with anti-Calreticulin immunofluorescence used to visualize the ER, indicating that those interactions took place in other cellular compartments. Those proteins were CH60, KCRB, and PGAM1 (Fig. S1). With four proteins, we observed no positive BiFC signal. Those proteins were ENOA, IF4A1, HS105, and ECHB (Fig. S2). Taken together, we were able to verify 60% or 6/10 of the tested interactions by BiFC. These data, to our knowledge, comprise the first study showing the PPIs of MANF in a cellular context as visualized by BiFC. What is more, although a systematic screening for MANF PPIs has not been conducted before, our findings are well in line with previously published data about MANF interacting with GRP170, GRP78 and PDIA6 (4, 36, 44). GRP78 as the major ER chaperone is involved in a variety of ER homeostatic processes. As such, we hypothesized that the neuronal survival-promoting action of MANF arises from its ability to interact with GRP78 and through this interaction counteract ER stress–related apoptosis.

### Direct interaction of MANF to GRP78 is cofactor-like, but not calcium-dependent

**Figure 4:**
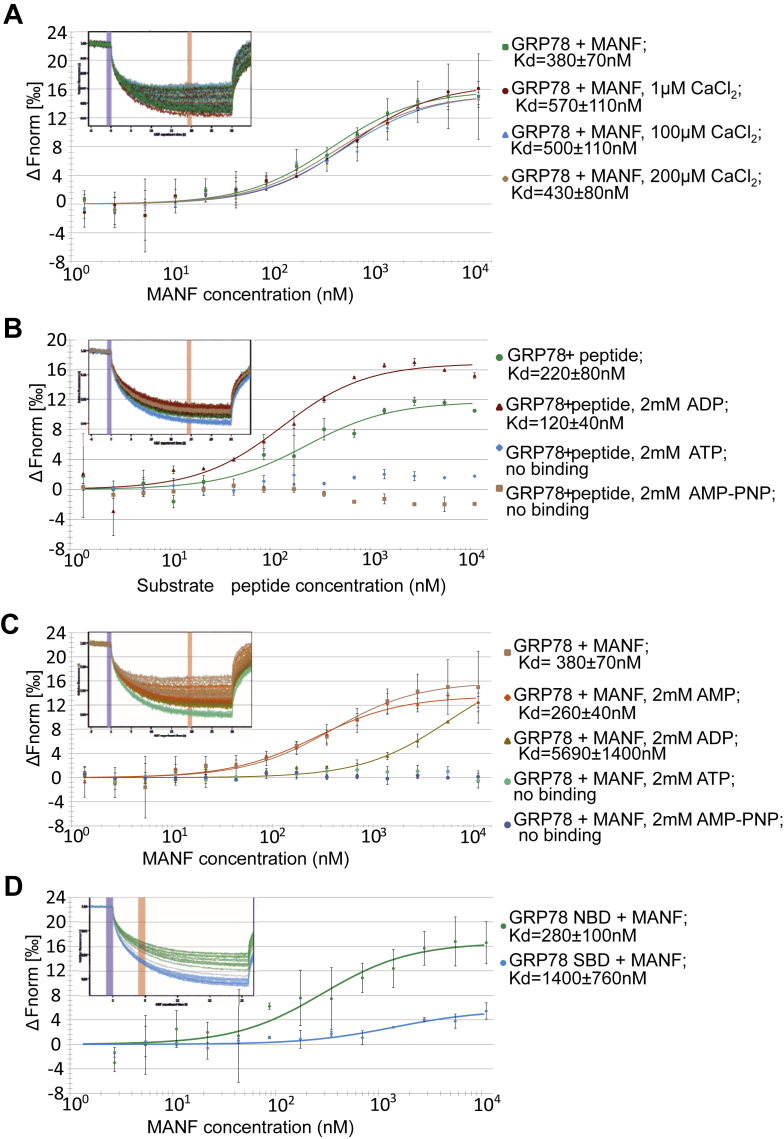
**MANF interacts with GRP78 in a direct, cofactor-like manner.***A*, microscale thermophoresis (MST) binding curves showing interaction between purified recombinant GRP78 that was fluorescently labeled and MANF, tested in at different CaCl_2_ concentrations. *B*, interaction between GRP78 and a substrate peptide (peptide), tested in the presence of ADP, ATP, or AMP–PNP. *C*, interaction between GRP78 and MANF, tested in the presence of AMP, ADP, ATP, or AMP–PNP. *D*, interaction between the nucleotide binding domain (NBD) and substrate binding domain (SBD) of GRP78 and MANF. The concentration of GRP78 or its variants was kept at a constant 20 nM in all experiments. All data were fitted using Nanotemper MO.Affinity Analysis v2.2.4 or v2.3 assuming binding with 1:1 stoichiometry. The plots show mean ΔFnorm values from n = 2 to 4 individual repeats per binding pair ± SD. Kd values ± error estimations calculated from the fits are shown in the figure legend. Normalized MST fluorescence traces of one representative experiment per binding pair are shown in the top left corners of the binding curve graphs. *Blue* and *red vertical margins* denote normalized fluorescence before and after induction of temperature gradient, respectively. GRP, glucose-regulated protein; MANF, mesencephalic astrocyte-derived neurotrophic factor.

Previous studies have found MANF to be a cofactor of GRP78 (43). In an effort to elucidate the MANF survival-promoting mechanism of action, we wanted to further study and verify this interaction and investigated this matter by using purified recombinant MANF and GRP78 proteins and MST. We show that MANF and GRP78 are, indeed, able to interact directly with a Kd = 380 ± 70 nM (Fig. 4*A*). It was also reported that the interaction between MANF and GRP78 is dependent on Ca^2+^ levels in the ER lumen (4). The ER is a major reservoir for intracellular Ca^2+^, and the levels of both cytosolic and intra-ER Ca^2+^ are tightly controlled as they regulate numerous cellular processes. However, the Ca^2+^-dependence of direct complex formation of purified recombinant MANF and GRP78 has not been investigated before. We performed an MST analysis of purified recombinant MANF and GRP78 proteins at several concentrations of Ca^2+^ and did not observe the Ca^2+^-dependency of the direct interaction as the Kd of the interaction did not change in the presence of 0, 1, 100, or 200 μM CaCl_2._ (Fig. 4*A*). We did, however, notice that the MANF–GRP78 protein complex started to precipitate at CaCl_2_ concentrations of 500 μM or more (data not shown). We thus conclude that, contrary to what has been previously suggested, direct interaction between MANF and GRP78 does not need nor is it dependent on the concentrations of Ca^2+^.

As the nature of MANF and GRP78 interaction has not been studied by MST, we next investigated whether MANF interacts with GRP78 as a substrate. First, as a control experiment, we measured the Kd of GRP78 and a substrate peptide interaction in the presence of different nucleotides. As a substrate, we used a well-characterized, IgG heavy chain-derived peptide (32). The Kd of GRP78 and substrate peptide interaction was 220 ± 80 nM in the absence of nucleotides and 120 ± 40 nM in the presence of ADP (Fig. 4*B*). The structures of the nucleotide-unbound (apo-) and ADP-bound GRP78 are very similar, explaining why they exhibit similar affinities toward a substrate peptide (32, 60). As expected, the GRP78-substrate peptide interaction was completely abolished by the addition of either ATP or its nonhydrolysable analog, AMP–PNP (Fig. 4*B*), demonstrating also that the recombinant GRP78 protein was active.

We then investigated the changes in MANF and GRP78 interaction in response to added nucleotides AMP, ADP, ATP, and AMP–PNP. In the presence of AMP, the Kd of MANF–GRP78 interaction was 260 ± 40 nM. As stated above, the Kd of GRP78 and MANF interaction was 380 ± 70 nM in the absence of nucleotides. Unlike in the case of GRP78 interaction with a substrate peptide, the interaction between GRP78 and MANF was weakened 15 times to 5690 ± 1400 nM upon the addition of ADP (Fig. 4*C*). Therefore, we concluded that folded, mature MANF is not a substrate for GRP78. Thus, it was surprising that the presence of ATP or AMP–PMP completely prevented the interaction of MANF and GRP78 (Fig. 4*C*).

We also tested MANF interaction with purified NBD and SBD domains of GRP78. MANF preferentially interacted with the NBD of GRP78. The Kd of this interaction was 280 ± 100 nM which is very similar to that of MANF and full-length GRP78 interaction, indicating that MANF mostly binds to the NBD of GRP78. We also detected some binding of MANF to the SBD of GRP78, but with a very small response amplitude and an affinity that was an order of magnitude weaker than that of both NBD and native GRP78 to MANF (Fig. 4*D*). The NBD of GRP78 did not bind the substrate peptide, whereas SBD did, indicating that the isolated SBD retains its ability to bind the substrates of full-length GRP78 (data not shown). These data are well in agreement with previously published data that MANF is a cofactor of GRP78 that binds to the N-terminal NBD of GRP78 (44), but in addition show that ATP blocks this interaction.

### MANF binds ATP *via* its C-terminal domain as determined by NMR

**Figure 5:**
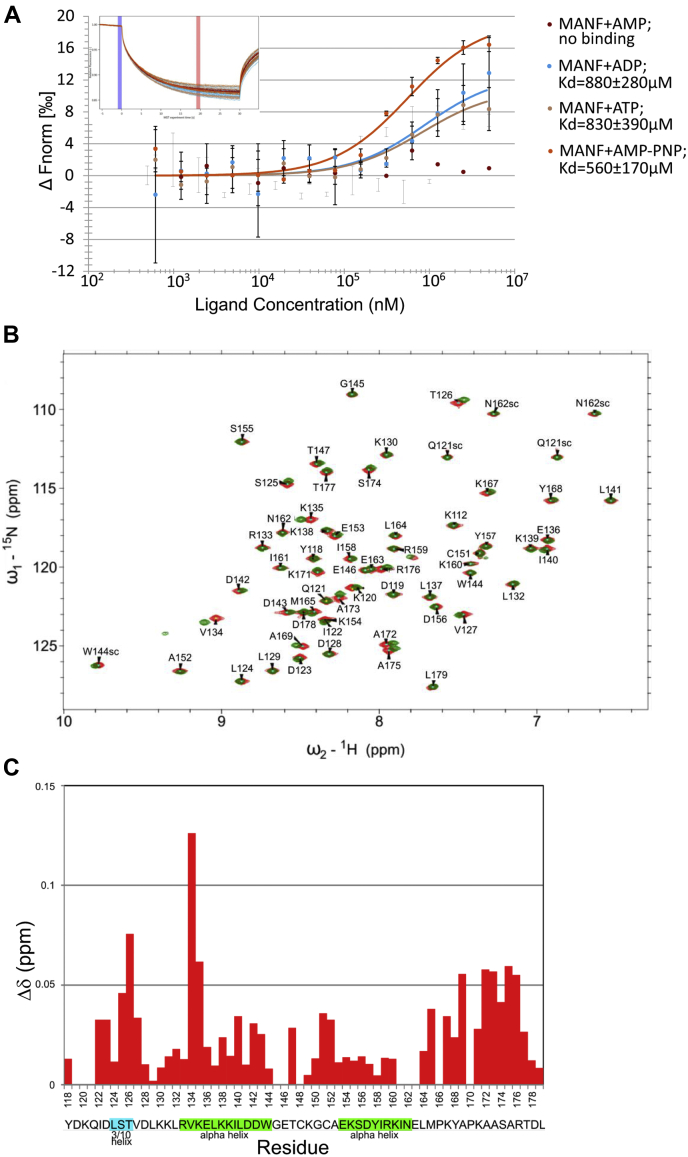
**MANF is a nucleotide-binding protein.***A*, MST binding curve of fluorescently labeled recombinant MANF and AMP, ADP, ATP, or AMP–PNP. All data were fitted using Nanotemper MO. Affinity Analysis v2.2.4 assuming binding with 1:1 stoichiometry. Plots show mean ΔFnorm values from two individual repeats per binding pair ± SD. Kd values ± error estimations calculated from the fits are shown as in the figure legend. Normalized MST fluorescence traces of one representative experiment per binding pair are show in the top left corner of the binding curve graphs. Blue and red margins denote normalized fluorescence before and after induction of temperature gradient, respectively. *B*, ^15^N-HSQC spectra of C-terminal domain of MANF (C-MANF) without ATP (*red*) and with ATP (*green*). Chemical shift assignments are included into the spectrum. Experiments were performed with C-MANF concentration of 0.1 mM and 1 mM ATP. *C*, normalized chemical shift perturbations (CSPs) observed in C-MANF because of ATP binding. The corresponding amino acid sequence and secondary structure elements of C-MANF are shown below the graph. MANF, mesencephalic astrocyte-derived neurotrophic factor; MST, microscale thermophoresis.

Because the conformations of apo-GRP78 and ADP-bound GRP78 are highly similar (32, 60), the observed highly different in Kd values of MANF interaction with GRP78 in the absence of nucleotides and presence of ADP (*i.e.*, 380 ± 70 nM and 5690 ± 1400 nM, respectively) could be explained only by changes in MANF conformation upon nucleotide addition. This might also explain the loss of GRP78–MANF interaction in the presence of ATP or AMP–PNP. As the nucleotide-binding ability of MANF has not been reported, we used MST to test it. Surprisingly, MANF did interact with ADP, ATP, and AMP–PNP with Kd-s of 880 ± 280 μM, 830 ± 390 μM, and 560 ± 170 μM, respectively, but not with AMP (Fig. 5*A*).

To study the interaction between MANF and ATP in more detail, we employed solution state NMR spectroscopy. NMR chemical shift perturbations (CSPs) are reliable indicators of molecular binding, even in the case of weak interaction. We added ATP to ^15^N-labeled full-length mature MANF in molar ratios 0.5:1.0, 1.0:1.0, and 10.0:1.0, which induced CSPs that increased in linear fashion upon addition of ATP (not shown). This is indicative of a fast dissociating complex, *i.e.*, weak binding which is in very good accordance with the results obtained from the MST studies. The ATP binding induced CSPs were localized to C-terminal domain of MANF (C-MANF), which we have previously shown to be an independently folding small structural module (15).

Next, we sought to study whether C-MANF is independently able to bind ATP in similar fashion to full-length MANF. Similar binding assay as in the case of full-length MANF was carried out for C-MANF, *i.e.*, using ATP in molar ratios of 0.5:1.0, 1.0:1.0, 10.0:1.0 (ATP:C-MANF). Identical CSPs were observed as in the case of full-length MANF. This indicates that the ATP binding site is located at the C-terminal domain of MANF. Figure 5*B* shows two-dimensional ^15^N, ^1^H correlation map of ^15^N-labeled C-MANF with 10-fold excess of ATP (green contours) and without *i.e.*, free protein (red contours). As can be observed from the CSP histogram ATP binding induced CSPs (Δδ) are small, exceeding 0.05 ppm only for 8 residues and 0.1 ppm only for amino acid V134 (Fig. 5*C*). These data correlate well with the results obtained from MST studies, *i.e.*, interaction with ATP is weak and imposes only minor conformational change in MANF. Interestingly, the ATP binding site of MANF, as indicated by evolutionarily fully or partially conserved amino acids V134 and K135 giving the biggest CSPs in NMR spectra, is directly adjacent to the R133 shown to play an important role in the binding of C-terminal domain of MANF to GRP78 (44).

**Figure 6:**
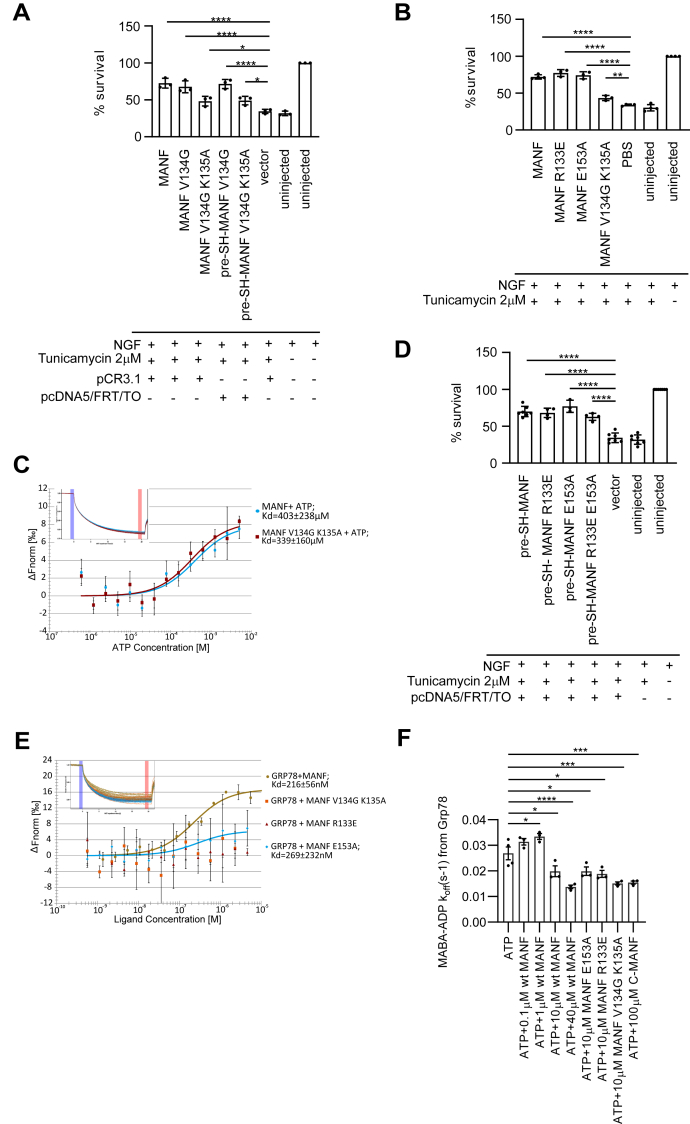
Mutating the ATP-binding site of MANF reduces its antiapoptotic activity regardless of the plasmid vector backbone. NGF-maintained embryonic SCG neurons were injected with the indicated MANF (*A*) expression plasmids either in pCR3.1 or pCDNA5/FRT/TO vectors or (*B*) wild-type or mutant proteins and treated with 2 μM tunicamycin. The number of surviving injected neurons was expressed as % of the number of living injected neurons counted 3 to 4 h after injection. Shown are the means of three independent experiments ± SD. Data of each experimental group were compared to pCR3.1 empty vector and uninjected or PBS, Tm treated controls using one-way analysis of variance (ANOVA) and Sidak’s multiple comparisons *post hoc* test. ∗, ∗∗ and ∗∗∗∗ denote *p*<0.05, *p*<0.01 and *p*<0.0001, respectively. The null hypothesis was rejected at *p* < 0.05. *C*, MST binding curve of fluorescently labeled recombinant wild-type and V134G K135A mutant of MANF and ATP. All data were fitted using Nanotemper MO.Affinity Analysis v2.3 assuming binding with 1:1 stoichiometry. Plots show mean ΔFnorm values from four to five individual repeats per binding pair ± SD. Kd values ± error estimations calculated from the fits are shown in the figure legend. Normalized MST fluorescence traces of one representative experiment per binding pair are show in the top left corner of the binding curve graphs. *Blue* and *red* margins denote normalized fluorescence before and after induction of temperature gradient, respectively. *D*, MANF R133E, E153A or R133E E153A mutants are antiapoptotic in neurons. NGF-maintained embryonic SCG neurons were injected with the indicated MANF expression plasmids and treated with 2 μM tunicamycin. The number of surviving injected neurons was expressed as % of the number of living injected neurons counted 3 to 4 h after injection. Shown are the means of three to six independent experiments ± SD. Data of each experimental group were compared with respective empty vector control using one-way analysis of variance (ANOVA) and Dunnett’s multiple comparison *post hoc* test. ∗∗∗∗ denotes *p* < 0.0001. The null hypothesis was rejected at *p* < 0.05. *E*, MST binding curves showing interaction between purified recombinant fluorescently labeled GRP78 and the indicated MANF variants. The concentration of GRP78 was kept at a constant 20 nM in all experiments. All data were fitted using Nanotemper MO. Affinity Analysis v2.3 assuming binding with 1:1 stoichiometry. The plots show mean ΔFnorm values from n = 2 to 3 individual repeats per binding pair ± SD. Kd values ± error estimations calculated from the fits are shown in the figure legend. Normalized MST fluorescence traces of one representative experiment per binding pair are shown in the top left corners of the binding curve graphs. *Blue* and *red vertical margins* denote normalized fluorescence before and after induction of temperature gradient, respectively. *F*, MABA–ADP release rates from 1.25 μM GRP78 in the presence of MANF or its variants at the indicated concentrations. Data of each experimental group were compared with ATP only group. Mean values ±SD of three to four independent experiments are shown. Ordinary one-way ANOVA and Dunnet’s multiple comparisons *post hoc* test, ∗*p* < 0.05, ∗∗*p*<0.01, ∗∗∗*p* < 0.001, ∗∗∗∗*p* < 0.0001. GRP, glucose-regulated protein; MANF, mesencephalic astrocyte-derived neurotrophic factor; MST, microscale thermophores; NGF, nerve growth factor; SCG, superior cervical ganglion; Tm, tunicamycin.

As a next step, we investigated the biological importance of amino acid residues V134 and K135 located in the ATP binding site of MANF, which was identified by NMR. For this, we used plasmid microinjection into cultured SCG neurons. Interestingly, the double mutation V134G K135A rendered MANF less active in promoting the survival of Tm-treated cultured SCG neurons, whereas single mutation V134G did not affect the survival promoting activity of MANF (Fig. 6*A*). These observations remained constant regardless of the vector backbone of MANF expression constructs used for neuronal microinjections. We noticed a similar effect when testing the survival-promoting properties of the purified recombinant MANF V134G K135A mutant protein in the Tm-treated SCG neurons (Fig. 6*B*). Namely, the MANF V134G K135A mutant protein was able to promote the survival of Tm-treated SCG neurons but was less active in comparison with wild-type MANF protein. We then used the purified recombinant MANF V134G K135A mutant protein to test the ATP-binding properties using MST. Surprisingly, the mutant was able to bind ATP similarly to the wild-type MANF (Fig. 6*C*). To conclude, we were able to create a MANF V134G K135A mutant with reduced antiapoptotic activity, but this mutant was still able to bind ATP. We suggest that the ATP-binding properties of MANF warrant further studies as these might have implications to the antiapoptotic activity of MANF as well as its ability to bind GRP78.

### Interaction with GRP78 is not required for the survival-promoting activity of MANF in neurons

Next, we investigated our hypothesis that MANF regulates UPR and cellular survival outcomes through its interaction with GRP78. MANF amino acid residues R133 and E153 were reported to be crucial for the GRP78 binding of MANF as mutating either of these amino acids rendered MANF unable to bind GRP78 (44). Consequently, we decided to test the biological importance of MANF–GRP78 interaction by generating MANF R133E and E153A mutants and investigating their ability to promote the survival of Tm-treated SCG neurons using plasmid microinjection (Fig. 1*A*). Unexpectedly, neither R133E nor E153A mutant exhibited loss of survival promoting activity as compared with the original SH-MANF expressing construct (Fig. 6*D*). We observed no changes in the survival promoting activity also for the MANF R133E and E153A double mutant (Fig. 6*D*). Additionally, we produced recombinant MANF proteins bearing R133E and E153A mutations and tested their survival promoting activity on Tm-treated SCG neurons using microinjection (Fig. 6*B*). Both MANF R133E and E153A proteins were as active as the wild-type MANF in promoting the survival of Tm-treated SCG neurons. Thus, these results fully supported those obtained by plasmid microinjection, confirming that R133E and E153A mutants are as active as the wild-type MANF in promoting neuronal survival. Using MST, we verified that neither R133E nor E153A MANF mutant protein was able to interact with GRP78, as reported (Fig. 6*E*) (44). Therefore, we conclude that interaction with GRP78 is not needed for the survival-promoting activity of MANF in this model of neuronal apoptosis. MANF V134G K135A double mutant protein, which was less active in promoting the survival of SCG neurons, was also unable to bind to GRP78 (Fig. 6*C*).

Finally, as MANF was recently reported to be an inhibitor of ADP release from GRP78 (44), we wanted to investigate how the activity of MANF V134G K135A double mutant compares to that of wild-type MANF in an MABA–ADP release assay. The release of fluorescent nucleotide analog MABA–ADP from Hsp70 chaperones to the aqueous environment can be monitored as decrease of fluorescence intensity (61). As controls, we included the MANF R133V and E153A mutant proteins previously shown to be inactive and a chemically synthesized polypeptide corresponding to the last 63 amino acids of MANF (C-MANF), shown to be active in the MABA–ADP release assay (44). C-MANF polypeptide at concentration 100 μM was actively inhibiting MABA–ADP release (Fig. 6*F*). Wild-type recombinant MANF protein was used at final concentrations of 0.1, 1, 10, and 40 μM. While MANF at concentrations 0.1 and 1 μM did not attenuate MABA–ADP release from GRP78, higher concentrations of 10 and 40 μM effectively inhibited nucleotide release from GRP78 (Fig. 6*F*). However, while the physiological concentrations of MANF vary depending on the tissue and cell type, we estimate that at its maximum, it is in a low micromolar range (62). Therefore, subsequently MANF mutants were tested at 10 μM concentrations. Surprisingly, all three tested MANF mutant variants V134G K135A, R133V and E153A were able to inhibit MABA–ADP release from GRP78 (Fig. 6*F*). This was especially unexpected for MANF R133V and E153A mutants as previously published study demonstrated their inability to inhibit MABA–ADP release from GRP78 (44). To conclude, we were able to demonstrate that MANF mutations V134G K135A, R133, or E153 negatively affect the interaction with GRP78, but our MABA–ADP release assay results indicate that their ability to inhibit nucleotide release from GRP78 remains unaffected. We therefore suggest that MANF has another, GRP78-independent mechanism for promoting neuronal survival. As both intracellularly and extracellularly applied MANF relies on the intactness of UPR signaling pathways, we suggest that the antiapoptotic mechanism of MANF might result in its ability to interact directly with the ER luminal domains of one or more UPR sensors (63).

## Discussion

Both the expression and secretion of MANF have been shown to increase in various cell types under conditions collectively known as the ER stress (4, 12, 19, 35, 36, 37). It has been proposed that chronic, unresolved UPR activation in pancreatic beta cells is the underlying cause of MANF-deficient mice developing diabetes. Thus, endogenous MANF is thought to exert its survival-promoting activity through regulating the UPR (39, 64). Interestingly, a recent paper reported that CNS-specific MANF knockout results in chronic activation of UPR, especially the IRE1 pathway, but no detectable neurodegeneration in the DA system (65). The perplexingly opposite cellular consequences of MANF-deficiency dependent UPR activation in pancreatic beta cells and neurons highlight the lack of understanding how MANF regulates UPR signaling. Therefore, to gain more knowledge about the MANF mechanism of action and the possible crosstalk between MANF and UPR signaling, we investigated the effect of MANF in two *in vitro* models of ER stress-caused apoptosis: mouse early postnatal SCG neurons treated with Tm and mouse embryonic DA neurons treated with Tg. We have shown previously that serum-deprived or etoposide-treated SCG neurons do not respond to exogenous MANF and thus used these neurons to study the effects of intracellularly expressed or added MANF (15). The survival-promoting effect of MANF overexpression in Tm-treated SCG neurons was abolished by inhibition of PERK or IRE1 signaling, suggesting that these UPR pathways are mediating intracellular MANF activity. Using the same paradigm, we injected recombinant MANF protein and observed a similar loss of MANF activity on PERK or IRE1 signaling inhibition. This is the first demonstration that intracellularly, at least in this particular neuronal ER stress-induced cell death model, MANF exerts its neuroprotective effects through modulating the UPR signaling occurring with the involvement of these ER transmembrane receptors.

We found that extracellularly added MANF had no effect on the survival of naïve mouse embryonic DA neurons but was able to increase neuronal survival when added simultaneously with Tg. Similarly was Tm-induced ER stress needed for intracellularly applied MANF to become effective in cultured SCG neurons. Thus, we conclude that the cellular counterparts mediating the uptake of MANF or its survival-promoting signaling become activated or expressed under conditions of ER stress. Like for intracellularly applied MANF, inhibiting IRE1 or PERK pathways reduced the survival-promoting effect of extracellularly added MANF. These data comprise, to our knowledge, a first evidence that intracellularly and extracellularly applied MANF rely on same intracellular pathways to promote survival of ER-stressed neurons. A recent study suggested that extracellular MANF relies on binding the lipid sulfatide for internalization and mediator of its cytoprotection (51). Sulfatide—a sulfoglycolipid synthesized in the ER and Golgi—can be found from the extracellular space and circulation among other locations and has a role in pathologies such as Parkinson’s disease, Alzheimer’s disease, and diabetes, to name a few (66, 67). Although outside the scope of this study, it would be interesting to investigate if interaction with extracellular sulfatide accounts for the stress-dependent survival-promoting activity of extracellularly added MANF in cultured DA neurons. In our recent article, we describe another candidate for MANF plasma membrane receptor. Interaction with NPTN was needed for MANF-dependent cell survival and regulation of inflammation. As NPTN is an ER stress–inducible protein, it could account for cultured DA neurons becoming responsive to MANF on ER stress (42). However, more studies are needed to investigate where exactly do the mechanisms of intracellularly and extracellulary applied MANF converge.

Because the activation of ATF6 differs from the activation of IRE1 and PERK pathways, studying the effect of MANF on ATF6 signaling-deficient systems might provide further understanding of how MANF affects the UPR signaling. Adding to these mechanistic insights, MANF added to the culture media of Tg-treated midbrain DA neuron cultures was able to downregulate UPR signaling of several UPR pathways as indicated by lower mRNA levels of s*Xbp1*, *Grp78*, and *Atf6* detected on MANF addition. This further strengthens the notion that MANF exerts it protective effects through modulating UPR signaling toward a mode more compatible with neuronal survival. While the fold change of *Chop* and *Atf4* transcripts did not reach statistical significance, a trend toward reduction could be observed also for these PERK pathway transcripts. It is, therefore, interesting to note that MANF can simultaneously regulate several, if not all, UPR pathways.

For a better understanding of the mechanism of action of MANF, we screened for its PPIs using AP-MS that has become the preferred high-throughput method for screening of interaction proteomes. Among other ER-located or ER-associated proteins, we also identified ER chaperones GRP78 and GRP170 as interactors of MANF in both cell lines studied. This finding is in agreement with and verifies other, previously published studies reporting these interactions (4, 36, 44). The conserved interactome of MANF consisted of 15 proteins and was relatively more enriched in ER-localized proteins. Additionally, GO term analysis of the conserved interactome indicated the involvement of the MANF conserved interactome in the ER homeostatic processes. Taken together, these data are well in line with the previously published data about the possible role of MANF in the ER homeostasis (19, 35, 36, 39, 44, 68, 69, 70).

Using BiFC, we were able to verify close to half (6/15) of MANF conserved PPIs. This is comparable to other studies where AP-MS has been followed by BiFC (45). All three tested ER-localized proteins GRP78, GRP170, and PDIA6 gave a BiFC signal with MANF. GRP78, GRP170, and PDIA6 have been identified to be a part of a large ER chaperone multiprotein complex, also termed an ER-localized multiprotein complex. Examples of other components of that complex are GRP94, ERdj3, PDIA1, PDIA2, PDIA4, and UGT1A1 (71). It was proposed that the purpose of such a large protein complex is to organize ER chaperones into a functional network allowing for efficient binding and folding of nascent proteins soon after their translocation into the ER lumen. In a study aiming to map the interaction network of an ER-localized chaperone GRP94 in the mouse preB leukemia cells, MANF was found to be one of the interacting proteins (72). We identified the MANF–GRP94 interaction in HEK293 cells, but not in INS1 cells. GRP94 and GRP170 were also, in addition to GRP78, identified as being in complex with MANF in a study published during the preparation of this manuscript (44). The same study identified MANF as a NEI of GRP78. This is well in agreement with our findings that MANF is a cofactor of GRP78 and forms complexes with several ER chaperones. What is more, we show for the first time the interaction of MANF with GRP78, GRP170, and PDIA6 in a cellular context.

UPR, as described above, is a set of dynamic signaling events aiming to control the ER protein-folding capacity by sensing and responding to changing protein loads in the ER. Accordingly, components of the large ER chaperone complex, including GRP78, GRP170, and PDIA6, have been shown to be involved in ER stress (23, 73, 74, 75, 76, 77, 78, 79, 80). Assuming that MANF is part of the ER-localized multiprotein complex, it is possible that it plays a role in regulating either the composition or activity of that complex in responding to changing substrate loads in the ER. This notion is supported by the observation that MANF, along with a few other know ER quality control proteins, GRP78, PDIA6, and GRP170 among those, was found to be specifically upregulated by misfolding-prone polypeptides (37). However, in case MANF is involved in the functioning of this complex through its interaction with GRP78 and possibly with other members of this complex, our data allow to conclude that the involvement of MANF in this protein complex might not be responsible for its role as an antiapoptotic factor in ER-stressed neurons.

Interestingly, GRP78, GRP170, and PDIA6 but also ribophorin 2 and CH60 were, among other proteins, found to form a complex with proinsulin, indicating a possible role in its folding and quality control (81). As those proteins were also found to be a part of the conserved interactome of MANF in our study, we hypothesize that MANF, too, is involved in the biosynthesis of insulin, possibly as a cofactor of GRP78 stabilizing the GRP78-insulin complex (39, 41, 44). The possible role of MANF in insulin biosynthesis needs, however, more studies.

In both cell lines studied, GRP78 was one of the most enriched proteins in MANF pull-downs. This is in agreement with previously published data showing that GRP78 co-immunoprecipitates with MANF (4).The role of GRP78 has been intensively studied in the maintenance of ER homeostasis and initiation of UPR signaling. Here, we hypothesized that the co-factor type interaction of MANF with GRP78 underlies its antiapoptotic function and decided to study the interaction of MANF with GRP78 in more detail. We used MST with purified recombinant MANF and GRP78 proteins to confirm that they indeed interact directly. However, contrary to what has been proposed before, we did not detect changes in the interaction Kd in response to changing Ca^2+^ levels (4). While the total Ca^2+^ concentration in the ER lumen has been estimated to be as high as 1 mM, the concentration of free Ca^2+^ is about 200 μM with the rest being bound by Ca^2+^-buffering proteins in the ER (82, 83). The 0 to 200 μM CaCl_2_ concentration range we tested is, therefore, a good representation of the free Ca^2+^ levels in the ER. It is possible that, *in vivo*, both GRP78 and MANF are part of a larger protein complex, such as the aforementioned large ER chaperone complex, containing additional proteins responsible for the dissociation of MANF from GRP78 in response to decreased ER luminal Ca^2+^ levels. Interestingly, recent studies have identified an inverse correlation between ER Ca^2+^ and ATP levels (84, 85). We therefore suggest that the increased dissociation of MANF from GRP78 and subsequently increased secretion under conditions of reduced ER Ca^2+^ observed by Glembotski *et al*. (4) is at least partly because of the increase of ATP concentration in the ER. The exact concentration of ATP within the ER lumen has remained unclear, but it has been estimated to be 1 to 10 mM (86, 87). It is also unclear what is the concentration of free ATP in the ER lumen, as a significant proportion of it appears to be bound and used by ATP-dependent proteins such as GRP78 and GRP94 (88). We propose that MANF exists in a dynamic equilibrium of association and dissociation from GRP78 in response to changing ATP levels in the ER lumen. The inverse correlation of ER Ca^2+^ and ATP would thus provide another regulatory layer of free or GRP78-bound MANF ratio. This is in a good agreement with our observation that although MANF does not bind GRP78 in a substrate-like manner, the complex between MANF and GRP78 dissociated in the presence of 2 mM ATP. Surprisingly, both MST and NMR spectroscopy showed the ability of MANF to bind ATP, with the site for ATP binding localized to the MANF C-terminal domain.

We hypothesized that the MANF antiapoptotic activity in neurons stems from its co-chaperone activity and, thus, tested the GRP78 binding-deficient MANF R133E and E153A mutants for their antiapoptotic activity in our cultured SCG neuron model (44). Unexpectedly, both mutants as well as the double mutant R133E E153A were able to promote the survival of SCG neurons as efficiently as wild-type MANF. This result was further verified by microinjecting either MANF R133E or E153A mutant proteins into the cytoplasm of cultured SCG neurons. These data show that the antiapoptotic activity of MANF, at least in this particular ER stress-related apoptosis model, is not dependent on its ability to bind to GRP78. This observation, therefore, suggests that MANF may have another mechanism, unrelated to GRP78 binding, for regulating ER stress-related apoptotic signaling. Consequently, we hypothesize that MANF is able to regulate the UPR signaling through directly or indirectly interacting with ER transmembrane UPR receptors—PERK, IRE1, or ATF6. By doing so, MANF is able to modulate the UPR signaling to a direction more favorable for neuronal survival. Our recent work allows us to propose that MANF regulates UPR signaling by directly binding to the ER luminal domains and regulating the activity of UPR receptors (63). In support of that hypothesis, we present evidence that the activity of IRE1 and PERK pathways are needed for the antiapoptotic activity of MANF in DA and SCG neurons.

We proposed that the nucleotide-binding activity of MANF is relevant for its function as a NEI of GRP78. We attempted to create a MANF variant deficient for nucleotide binding by mutating the V134 and K135 amino acid residues located in the C-terminal domain of MANF. While mutating the residue V134 alone did not have an effect on the antiapoptotic properties of MANF, the V134G K135A double mutant was less active in promoting the SCG neuron survival when either microinjected into the neurons as a plasmid or into the cytoplasm as recombinant protein. Much to our surprise, the MANF V134G K135A double mutant protein was still able to bind ATP in the MST assay. This suggests that the reduction in the antiapoptotic activity we observed with this mutant did not result from altered ATP-binding properties like we initially thought. Interestingly, while the V134G-K135A mutation did not affect the ATP-binding properties of MANF, it abolished the interaction with GRP78. This indicates that this region in the C-terminal domain of MANF plays an important role in mediating the MANF–GRP78 interaction. More studies are, however, needed to elucidate how this region of C-terminal MANF protein participates in the antiapoptotic function of MANF as well as the exact role of ATP binding in MANF biological activity.

We used MST to verify a previously published result that MANF binds GRP78 in a cofactor-like manner (44). We were, however, unable to confirm the finding that abolishing the ability of MANF to interact with GRP78 renders the former unable to inhibit ADP release from GRP78. All the MANF mutants tested in MABA–ADP release assay were deficient in GRP78 binding, but successfully attenuated MABA–ADP release from GRP78. Therefore, we hypothesize that the reported ability of MANF to act as a NEI for GRP78 is, at least partially, an effect of MANF scavenging the ATP in the nucleotide exchange reaction, thus compromising the availability of ATP for GRP78 to undergo a nucleotide exchange. This hypothesis warrants more studies with ATP-binding deficient MANF mutants.

In summary, we show for the first time that the neuroprotective mechanism of both intracellularly and extracellularly applied MANF depend on the activity of PERK and IRE1 UPR pathways. Using DA neuron cultures, we report that MANF is able to downregulate the transcript levels of components of several UPR pathways, but especially those of IRE1 and ATF6. We have identified several previously unknown interacting proteins for MANF as well as confirmed the previously reported cofactor-type interaction with GRP78 (4, 44). GO term enrichment analysis of the MANF conserved interactome point toward the involvement of MANF in regulating the cellular protein homeostasis. However, contrary to previously published work, our data suggest that MANF might not be a classical NEI of Hsp70 chaperones as the ability of MANF to regulate nucleotide release and binding by GRP78 was not altered by abolishing the interaction between MANF and GRP78. Unexpectedly, functional analysis of GRP78-binding deficient mutants of MANF indicated that interaction with GRP78 is not required for the survival-promoting activity of MANF in neurons. Interestingly, through its C-terminal domain, MANF itself is able to bind nucleotides such as ATP and ADP, as shown by MST and solution state NMR. What is more, mutating the V134 and K135 at the core of the ATP-binding site of MANF reduced the survival promoting activity of MANF in an ER-stress induced neuronal apoptosis model, without compromising the ability of MANF to bind ATP. Although the observed conformational changes of MANF upon nucleotide binding are small, it is possible that these reduce the ability of MANF to bind GRP78 or other UPR signaling-related proteins in the ER. Unfortunately, we did not succeed in generating an ATP-binding deficient mutant of MANF and were thus unable to study the role nucleotide binding has in the biological function of MANF. However, we hypothesize that the role of MANF as a NEI for GRP78 relies on its ability to bind and scavenge nucleotides, rather than its direct interaction with the chaperone. What is more, we propose that the neuroprotective effects of MANF relies on its ability to modulate several UPR pathways by interacting with the ER luminal domains of UPR sensors, thus steering them toward UPR activation levels or mode more compatible with neuronal survival.

## Experimental procedures

### Recombinant MANF proteins

Recombinant human MANF protein was produced from a CHO-derived cell line using the QMCF technology as has been described before (P-101-100, Icosagen Ltd) (89). The MANF R133E, E153A, and V134G K135A mutant recombinant proteins were made to order by Icosagen using the same technology. Briefly, codon-optimized cDNAs were cloned to pQMCF-T expression vectors which were then transiently transfected to CHO-derived protein production cell line. Proteins were captured and purified from the cell culture media using 5 ml Q FF followed by 1 ml SP HP, buffer was exchanged into PBS pH 7.4 by size exclusion chromatography. Protein purity was verified by SDS-PAGE with Coomassie staining and immunoblotting using rabbit anti-MANF antibody (310-100, Icosagen Ltd).

### Plasmids for MANF expression and for the generation of doxycycline inducible cell lines

To generate the MANF Gateway compatible entry vector, pCR3.1 MANF (90) was cloned into pENTR221 vector using Gateway entry clone generation by PCR (Invitrogen, Thermo Fisher Scientific). In addition, we amplified the Twin-Strep- and a hemagglutinin- (SH) tag from the pcDNA5/FRT/TO/SH vector and inserted it between the sequences coding for signal peptide (pre) and mature regions of human MANF to generate the pre-SH-MANF entry clone. Finally, pre-SH-MANF and GFP were cloned into pcDNA5/FRT/TO/cSH-destination vector (46) using Gateway LR cloning (11791020, Invitrogen, Thermo Fisher Scientific). These constructs were further used for activity testing and generating stable isogenic doxycycline-inducible pre-SH-MANF and GFP cell lines.

pcDNA5/FRT/TO pre-SH-MANF R133E, E153A, R133E E153A, V134G, and V13G K135A and pCR3.1 MANF V134G and V134G K135A mutants were generated using site-directed inverse PCR mutagenesis and pcDNA5/FRT/TO pre-SH-MANF or pCR3.1 MANF, respectively, as templates.

We used the SignalP 4.0 signal peptide prediction algorithm to check that the MANF presequence followed by an SH-tag would still be recognized as an ER signal peptide and thus cleaved by mammalian signal peptidase complex (91).

### Plasmids for BiFC

pCE-BiFC-VC155 (CV) and pCE-BiFC-VN173 (NV) were a gift from Chang-Deng Hu (Addgene plasmids #22020 and #22019). pEZYflag and pEZYmyc-His were a gift from Yu-Zhu Zhang (Addgene plasmids #18700 and #18701). Gateway destination vectors for BiFC for N- and C-terminal tagging with Venus fluorescent protein fragments (pEZY BiFC N NV, pEZY BiFC N CV, pEZY BiFC C NV and pEZY BiFC C CV) were generated by PCR by replacing the flag or myc-His sequences from pEZYflag or pEZYmyc-His with VC155 or VN173 sequences from the respective plasmids.

The following Gateway entry clones were from the Genome Biology Unit Core Facility (Research Programs Unit, Faculty of Medicine, HiLIFE, University of Helsinki, Biocenter Finland): HSPA5 (GRP78) without stop (DQ895368), PDIA6 without stop (DQ894369), JUN without stop (DQ896432), FOS without stop (DQ893444), MYC without stop (DQ894085), MAX without stop (JF432558). Shown is the Genbank accession number and the presence or absence of a translation stop-codon to indicate subsequent N- or C-terminal fusion, respectively, with a Venus fragment.

pcDNA3.1 HYOU1 (Grp170) was a gift from Linda Hendershot and was used to clone the sequence corresponding to full-length HYOU1 into pENTR221 vector following manufacturer’s instructions for generating Gateway entry clones by PCR and Gateway BP clonase reaction (Thermo Fisher Scientific).

pENTR221 pre-C-Venus-MANF and pENTR221 pre-N-Venus MANF were generated by amplifying the sequences corresponding to VC155 and VN173 from the respective BiFC destination vectors and inserting those between the sequences coding for signal peptide (pre) and mature regions of human MANF into the pENTR221 MANF with stop codon at the end of MANF reading frame. The corresponding BiFC expression plasmids (pEZY BiFC pre-NV-MANF and pEZY BiFC pre-CV-MANF) were made by LR clonase recombination reaction of pENTR221 pre-NV-MANF and pENTR221 pre-CV- MANF into pEZY Myc-His destination vector.

### Neuronal cell culture and microinjection

Culture of mouse SCG sympathetic neurons and microinjection of these neurons was performed as described earlier (15). Briefly, the neurons of postnatal day 1 to 2 NMRI strain mice were grown 6 DIV on polyornithine-laminin (P3655 and CC095, Sigma-Aldrich)–coated dishes with 30 ng/ml of 2.5 S mouse nerve growth factor (G5141, Promega). The nuclei were then microinjected with the expression plasmid for full-length (FL)-MANF together with a reporter plasmid for enhanced green fluorescent protein (EGFP), at concentration of 10 ng/μl in each experiment. For protein microinjection, recombinant full length (FL-) MANF protein (P-101-100, Icosagen, Estonia) or the indicated mutant proteins in PBS at 200 ng/μl were microinjected directly into the cytoplasm together with fluorescent reporter Dextran Texas Red (MW 70,000 Da) (D1864, Invitrogen, Molecular Probes) that facilitates identification of successfully injected neurons. Next day, Tm (2 μM) (ab120296, Abcam) was added and living fluorescent (EGFP-expressing or Dextran Texas Red-containing) neurons were counted 3 days later and expressed as percentage of initial living fluorescent neurons counted 2 to 3 h after microinjection. PERK signaling inhibitor GSK2606414 (516535, Merck Millipore) or IRE1 signaling inhibitor 4μ8C (4479, Tocris Bioscience) were used when indicated.

### Primary cultures of midbrain dopamine neurons and MANF treatment

The midbrain floors were dissected from the ventral mesencephali of 13-days-old NMRI strain mouse embryos. The tissues were incubated with 0.5% trypsin (103139, MP Biomedical) in HBSS (Ca^2+^/Mg^2+^-free) (14170112, Invitrogen) for 20 min at 37 °C, then mechanically dissociated. Cells were plated onto the 96-well plates coated with poly-L-ornithine (Sigma-Aldrich). Equal volumes of cell suspension were plated onto the center of the dish. The cells were grown for 5 days with 100 ng/ml MANF (P-101-100, Icosagen). GDNF (100 ng/ml) (P-103-100, Icosagen) or a condition without any neurotrophic compound added were used as positive and negative controls, respectively, where indicated.

After growing 5 days, the neuronal cultures were fixed and stained with mouse anti-tyrosine hydroxylase (TH) antibody (MAB318, RRID: AB_2201528, Millipore Bioscience). Images were acquired by CellInsight high-content imaging equipment (Thermo Fisher Scientific). Immunopositive neurons were counted by CellProfiler software (92), and the data were analyzed by CellProfiler Analyst (93) software. The results are expressed as % of cell survival compared with GDNF-maintained neurons.

For DA neuron survival experiment with IRE1 or PERK inhibitors, embryonic day (E)13.5 DA neurons were cultured without any trophic factors for 5 days, then treated 3 days with Tg (20 nM) or Tg and MANF or Tg and MANF and the indicated inhibitors. After 3 days of treatment, the cells were fixed, stained with anti-TH antibody, and imaged as described above. The results are expressed as percentage of TH-positive cell survival as compared to the non-Tg treated condition.

### RNA isolation, reverse transcription, and quantitative PCR

Midbrain dopaminergic neurons were isolated and cultured for 5 to 7 days as described and then treated with Tg (100 nM) (T7458, Molecular Probes). Recombinant MANF protein (100 ng/ml) (P-101-100, Icosagen) was added to the cultures at the same time. After 24 h, RNA from cultured cells was isolated by TriReagent (RT118, Molecular Research Center) according to manufacturer's instructions. RNA was reverse transcribed to cDNA with RevertAid Premium Reverse Transcriptase (EP0441, Fermentas UAB, Thermo Fisher Scientific). Quantitative PCR was performed using LightCycler 480 SYBR Green I Master (04887352001, Roche Diagnostics GmbH) and Roche LightCycler 480 Real-Time PCR System (Roche Diagnostics GmbH). The expression levels were normalized to the levels of β-actin in the same samples. Primers used in quantitative PCR were synthetized using previously published sequences (39) as below:

*Xbp1*_total: 5′-CACCTTCTTGCCTGCTGGAC-3′, 5′-GGGAGCCCTCATATCCACAGT-3′

*Xbp1*_spliced: 5′-GAGTCCGCAGCAGGTG-3′, 5′-GTGTCAGAGTCCATGGGA-3′

*Atf4*: 5′-ATGGCCGGCTATGGATGAT-3′, 5′-CGAAGTCAAACTCTTTCAGATCCATT-3′

*Chop*: 5′- CCAACAGAGGTCACACGCAC-3′, 5′-TGACTGGAATCTGGAGAGCGA-3′

*Atf6a*: 5′-GGACGAGGTGGTGTCAGAG-3′, 5′-GACAGCTCTTCGCTTTGGAC-3′

*Grp78*: 5′-ACCCTTACTCGGGCCAAATT-3′, 5′-AGAGCGGAACAGGTCCATGT-3′

### Cell line generation and culture

Flp-In 293 T-REx cells (HEK293 parental, Invitrogen) containing a single genomic FRT site and stably expressing the Tet repressor were grown in Dulbecco’s modified Eagle medium (Sigma-Aldrich) supplemented with 10% fetal bovine serum (Gibco, Thermo Fischer Scientific) and 50 μg/ml Normocin (ant-nr-2, InvivoGen).

Flp-In INS1 #5-3.19 cells (INS1 parental) were a gift from G.Ryffel and S.Senkel and have been described elsewhere (55). INS1 parental cells were grown in RPMI-1640 media supplemented with 10% FBS, 1 mM sodium pyruvate (S8636, Sigma-Aldrich), 10 mM HEPES pH 7.2, 2 mM L-glutamine (25030-024, Gibco, Thermo Fisher Scientific), and 50 μM beta-mercaptoethanol (31350-010, Gibco, Thermo Fisher Scientific). For targeted integration of MANF, both HEK293 and INS1 parental cells were transfected with pcDNA5/FRT/TO pre-SH-MANF or pcDNA5/FRT/TO GFP-SH expression plasmids and pOG44 vector (Invitrogen) for co-expression of the Flp-recombinase using FugeneHD (E2311, Roche) transfection reagent. Two days after transfection, the stable cell lines were selected with 50 μg/ml Hygromycin-B Gold (ant-hg-1, InvivoGen) for 2 weeks.

### Affinity purification

Transgenic HEK293 pre-SH-MANF, HEK293 GFP-SH, INS1 pre-SH-MANF, and INS1 GFP-SH cells were plated on 15 cm cell culture plates (10 plates for each cell line). Transgene expression was induced by adding media containing 1 μg/ml doxycycline (D9891, Sigma-Aldrich) for 24 h. At approximately 70%, confluency cells were induced with 1 μg/ml doxycycline (D9891, Sigma-Aldrich) for 24 h. For each purification sample, cells from 5 × 15 cm plates were washed with ice-cold PBS (supplemented with 0.1 mM MgCl_2_ and 0.1 mM CaC_2_), harvested in PBS-1 mM EDTA, snap frozen, and stored at −70 °C until purification. For affinity purification, pellet (approximately 1 × 10^8^ cells) was lysed in 3 ml of HENN lysis buffer (50 mM Hepes-NaOH, pH 8.0, 5 mM EDTA, 150 mM NaCl, 50 mM NaF, 0.5% NP40, 1 mM PMSF, 1.5 mM Na_3_VO_4_, and 0.1× protease inhibitor cocktail (P8340, Sigma-Aldrich) on ice for 10 min and centrifuged twice at 16,000*g* for 15 min at 4 °C to remove any insoluble material. The cleared lysates were then loaded on spin columns (732-6008, Bio-Rad Laboratories) containing 200 μl Strep-Tactin beads (2-1201-002, IBA GmbH). The beads were washed three times with 1 ml ice-cold HENN lysis buffer, followed by three washes with 1 ml HENN buffer without detergent and inhibitors. Proteins were eluted with 1 mM D-biotin (29129, Thermo Fisher Scientific) in 600 μl of HENN buffer without detergent and inhibitors.

### Preparation for mass spectrometry analysis

Eluates were neutralized with 100 mM ammonium bicarbonate (NH_4_HCO_3_) and the peptide cysteine bounds were reduced and alkylated with 5 mM Tris (2-carboxyethyl)phosphine and 10 mM iodoacetamide, respectively. Proteins were digested into peptides by adding 1 μg of sequencing grade trypsin (Promega) and after overnight incubation at 37 °C, samples were quenched with 10% TFA and purified with C18 Micro SpinColumns according manufacturer’s instructions (The Nest Group). Finally, samples were re-dissolved in 30 μl buffer A (0.1% trifluoroacetic acid and 1% acetonitrile in LC-MS grade water) for the LS-MS analysis.

### Mass spectrometry analysis

The LC-MS analysis was performed on Orbitrap Elite hybrid mass spectrometer coupled to EASY-nLC II –system using the Xcalibur, version 2.7.0 SP1 (Thermo Fisher Scientific). A total of two biological replicates and two technical replicates were used for each cell line. An exception to this was the INS1 pre-SH-MANF cell line where one of the biological replicates was analyzed in only one technical replicate. The tryptic peptide mixture (4 μl) was first loaded into a C18-packed precolumn (EASY-Column 2 cm × 100 μm, 5 μm, 120 Å, Thermo Fisher Scientific) in 10 μl volume of buffer A and then to C18-packed analytical column (EASY-Column 10 cm × 75 μm, 3 μm, 120 Å, Thermo Fisher Scientific). To separate the peptides, a 60-min linear gradient at the constant flow rate of 300 nl/min from 5 to 35% of buffer B (98% acetonitrile and 0.1% formic acid in MS grade water) was used. Analysis was performed in data-dependent acquisition: one high resolution (60,000) FTMS full scan (m/z 300–1700) was followed by top20 CID-MS2 scans in ion trap (energy 35). Maximum FTMS fill time was 200 ms (Full AGC target 1,000,000), and the maximum fill time for the ion trap was 200 ms (MSn AGC target of 50,000). Precursor ions with more than 500 ion counts were allowed for MSn. To enable the high resolution in FTMS scan, preview mode was used.

### Protein identification and quantification

Proteins were identified and MS1 quantified using Andromeda search engine and MaxQuant proteomics software (version 1.5.5.1) (94). Thermo.raw files were searched against the human or rat component of the UniProt-database complemented with trypsin, BSA, GFP, and tag sequences (human: release 2016_1; 20,149 entries, rat: release 2016_10; 7973 entries). In addition, rat database was completed with human MANF sequence. Carbamidomethylation (+57.021464 Da) of cysteine residues was used as static modification and oxidation (+15.994491 Da) of methionine as dynamic modification. A total of two missed cleavages were allowed. Error tolerances on the precursor and fragment ions were ±4.5 ppm and ±0.5 Da, respectively. Peptide FDR was set to <0.05.

### MS data filtering and analysis

Further data filtering and analysis were performed using the Perseus software platform (version 1.5.5.3) (95). The HEK293 data set consisted of proteins detected from HEK293 GFP-SH and HEK293 pre-SH-MANF cell lines. The INS1 data set consisted of proteins detected from INS1 GFP-SH and INS1 pre-SH-MANF cell lines. Briefly, for normal distribution, the intensity values from MaxQuant software were base2 logarithmized. Missing values were imputed by random selection from a normal distribution shifted from the measured data distribution toward the lower intensity values (down shift 1.8 and width 0.3 of standard deviations). Proteins represented by less than three valid values of the quadruplicate runs per bait (triplicate runs for pre-SH-MANF in INS1 cells) were excluded from further analysis. Ribosomal and keratin proteins were manually excluded from further analysis as these have been commonly identified as nonspecific interactor proteins (96). For visualizing and filtering of high confidence interactors in Perseus, we used the Volcano plot plugin which is a combined function of permutation-based FDR-controlled two-sample *t*-test and a scatter plot. The *p*-values were adjusted to 0.1% FDR, and the scaling factor s0 was set to 2. Interaction networks were visualized with Cytoscape software (version 3.5.1) (97). For functional annotations and enrichment analysis, we used the Panther database (version 11) (98). CORUM database was used to identify enriched protein complexes in the MANF interactomes (99, 100).

### Bimolecular fluorescence complementation assay

For BiFC, HEK293 cells were plated onto Poly-D-Lysine (P0899, Sigma-Aldrich) coated coverslips 48 h before transfection. Cells were co-transfected with the indicated pEZY BiFC C-Venus and N-Venus plasmids using the JetPEI transfection reagent (101, Polyplus Transfection) according to the manufacturer’s instructions. Twenty to 24 h after transfection, the cells were fixed with 4% PFA, permeabilized with 0.1% Triton X-100 and stained with rabbit anti-calreticulin (1:500, RRID:AB_303402, ab2907, Abcam), and goat anti-rabbit Alexa Fluor 568 (1:1000, A11011, RRID:AB_143157) from Thermo Fisher Scientific. Hoechst 33342 (H1399, Invitrogen) was used for nuclear counterstaining and ProLong Diamond Antifade Mountant (P36965, Thermo Fisher Scientific) for mounting.

### Imaging

All images were taken using the LSM 700 (Carl Zeiss) confocal microscope, LCI Plan-Neofluar 63×/1.30 glycerol immersion objective at room temperature, and Zen Black acquisition software (Carl Zeiss AG). Image analysis was done using the Zen Blue Lite (Carl Zeiss), PHOTO-PAINT and CorelDraw programs from the CorelDRAW Graphics Suite 2017. Postimaging processing was done using Corel PHOTO-PAINT 2017 using the brightness/contrast/intensity adjustment settings equally for images from the same imaging series.

### Microscale thermophoresis

The binding affinities of recombinant protein interactions were analyzed by microscale thermophoresis using Monolith NT.115 Instrument (NanoTemper Technologies GmbH). All measurements were performed at 25 °C, at 20% or medium MST power, depending on the version of Monolith control software used. The LED power was set to 50% and 100% for labeled MANF and GRP78, respectively.

The recombinant hamster GRP78 protein was purified as described before (101, 102). The generation and purification of GRP78 NBD have also been described before (103). The SBD of GRP78 was a kind gift from M. Ali and has been described previously (24). GRP78 and its variants were labeled through their N-terminal His-tags using Monolith His-Tag Labeling RED-tris-NTA kit (L008, NanoTemper Technologies GmbH) or Monolith His-Tag Labeling Kit RED-tris-NTA second Generation (MO-L018, NanoTemper Technologies GmbH), respectively. Recombinant human MANF protein (P-101-100, Icosagen) and its variants (custom production, Icosagen) were labeled using the amine-reactive Monolith Protein Labeling Kit RED-NHS kit (L001, NanoTemper Technologies GmbH). Removal of free, unreacted dye from MANF after the labeling reaction was done using Zeba Spin Desalting Columns (Thermo Fisher Scientific) according to the manufacturer’s instructions. Final concentrations of labeled proteins in interaction measurements were kept constant at 20 nM for GRP78 and its variants and at 50 nM for MANF. The ligands were titrated in 2-fold dilutions with indicated concentrations. All experiments were done using Monolith NTT premium-coated capillaries (K005, NanoTemper Technologies GmbH), 12 to 14 capillaries in each measurement. Two to five independent measurements were done for each binding pair.

All experiments were done in MST buffer (10 mM Na-phosphate buffer, pH7.4, 1 mM MgCl_2_, 3 mM KCl, 150 mM NaCl, and 0.05% Tween-20) with ATP (A2383, Sigma-Aldrich or R0441, Thermo Fisher Scientific), ADP (A5285, Sigma-Aldrich), AMP (01930, Sigma-Aldrich), AMP-PNP (10102547001, Sigma-Aldrich), or CaCl_2_ added where indicated.

The small, CH1-derived peptide (HTFPAVL) (custom peptide synthesis by Pepscan, the Netherlands) used as a model substrate for GRP78 has been described before (32).

### NMR

Expression and purification of ^15^N labeled full-length MANF and C-MANF have been described earlier (15). Full-length MANF and C-MANF were in 20 mM sodium phosphate buffer, pH 6.5, 50 mM NaCl and containing 5% (v/v) D_2_O for NMR deuterium lock signal. All NMR experiments were carried out at 25 °C, using Bruker AVANCE III HD 800 NMR spectrometer, equipped with cryogenically cooled TCI ^1^H, ^13^C, ^15^N probehead with z-gradient coil. Nucleotide-binding assay was carried out by adding ATP in molar ratios 0.5, 1.0, and 10.0 to either 100 μM full-length MANF or 100 μM C-MANF and monitoring ATP binding-induced CSPs in MANF using two-dimensional heteronuclear single quantum coherence, ^15^N-HSQC, experiment. CSPs (Δδs) were calculated with equation δ = (ΔδH (2) + (0.154 × ΔδN) (2))^1/2^.

### MABA–ADP release assay

Assaying the release of fluorescent ADP-analog MABA–ADP was done essentially as has been published before (44). Briefly, 5 μM GRP78 was mixed with MABA–ADP (NU-893-MNT, Jena Bioscience) in equal volume and equimolar concentration and incubated for 3 h at 30 °C while protected from light. Nucleotide exchange solutions contained 250 μM ATP (R0441, Thermo Fisher Scientific) and proteins as indicated. For MABA–ADP release assay, equal volumes of GRP78-MABA–ADP and nucleotide exchange solutions were mixed and immediately transferred to quartz cuvettes (Z802336, Hellma Analytics, Sigma Aldrich). Release of MABA–ADP from GRP78 was monitored as the decrease of fluorescence at excitation 360 nm and emission 420 nm, measured using a Perkin Elmer LS55 fluorescence spectrometer. Final concentrations of reaction components: GRP78 1.25 μM, MABA–ADP 1.25 μM, ATP 125 μM, and MANF as indicated. Reaction buffer was 50 mM HEPES-KOH pH7.4, 100 mM KCl, 10 mM MgCl_2_. MABA–ADP release curves were normalized after subtracting background (buffer-only measurement), curves were fitted, and MABA–ADP release rates calculated using the one-phase exponential decay function in Graphpad Prism 8. A total of three to four independent experiments were performed to determine the mean k_off_ rates of MABA–ADP release.

## Data availability

Original thermo.raw-files MS analysis have been uploaded to MassIVE and made publicly accessible. The project ID is MSV000086342 and the files can be downloaded from ftp://massive.ucsd.edu/MSV000086342/.

## Conflict of interest

The authors declare that they have no conflicts of interest with the contents of this article. Mart Saarma and Päivi Lindholm are inventors in a MANF-related patent owned by Herantis Pharma Plc.
