# Supplementary material for: The cytoprotective protein MANF promotes neuronal survival independently from its role as a GRP78 cofactor
Source: J Biol Chem. 2021 Jan 15;296:100295. doi: 10.1016/j.jbc.2021.100295 (PMC7949057; doi:10.1016/j.jbc.2021.100295)
Supplement: Supplemental Figures S1–S2 [file mmc4.pdf]

The cytoprotective protein MANF promotes neuronal survival independently from its role as a GRP78 cofactor (SUPPORTING DATA)

Ave Eesmaa[1], Li-Ying Yu[1], Helka Göös[1], Kristofer Nõges[1], Vera Kovaleva[1], Maarit Hellman[2], Richard Zimmermann[4], Martin Jung[4], Perttu Permi [2,3], Markku Varjosalo[1], Päivi Lindholm [1]*, Mart Saarma[1]*

From the [1]Institute of Biotechnology, HiLIFE, University of Helsinki, Finland; [2] Department of Chemistry, Nanoscience Center, University of Jyvaskyla, FI-40014, Jyvaskyla, Finland; [3] Department of Biological and Environmental Science, Nanoscience Center, University of Jyvaskyla, FI-40014, Jyvaskyla, Finland; [4] Medical Biochemistry and Molecular Biology, Saarland University, 66421 Homburg, Germany

Running title: *MANF-GRP78 interaction not required to rescue neurons*

*Shared senior authors and to whom correspondence should be addressed. E-mail: paivi.pulkkila@helsinki.fi and mart.saarma@helsinki.fi

Supporting figure S1

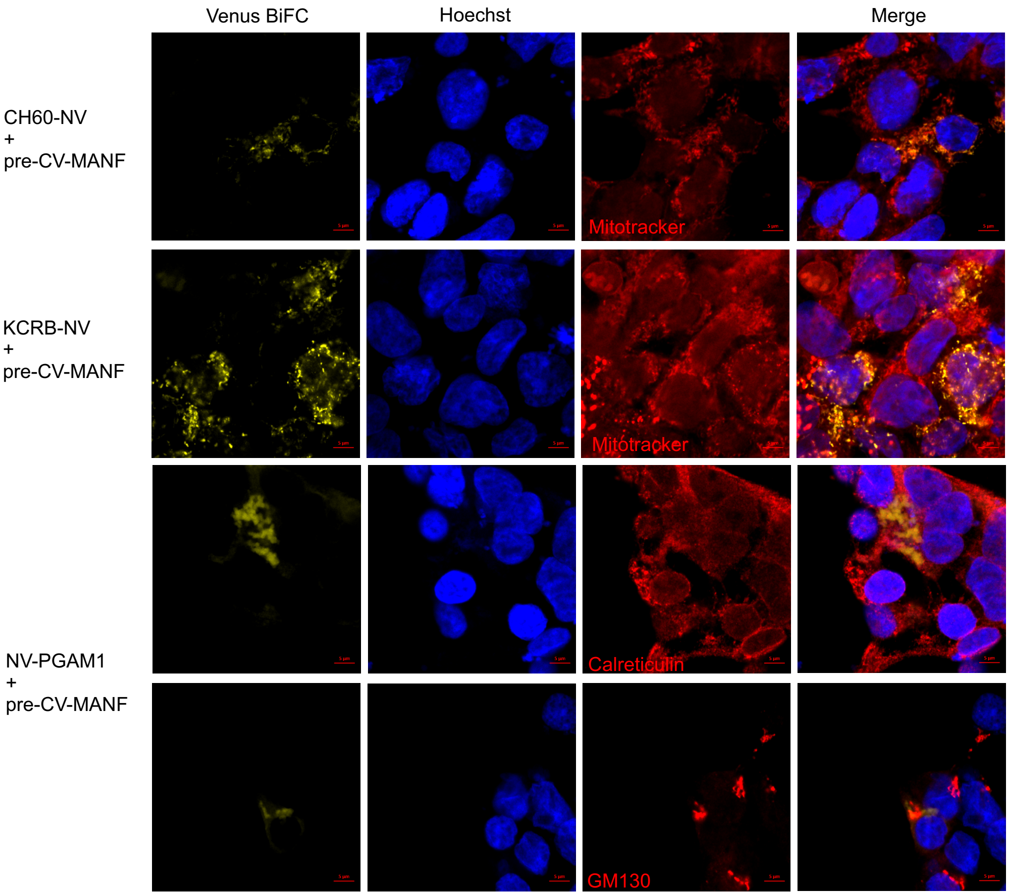

Supporting figure S1. Positive BiFC signals between MANF and proteins from the MANF conserved interactome, observed in cellular compartments other than the ER. Red scale bars denote 10 μm.

Supporting figure S2

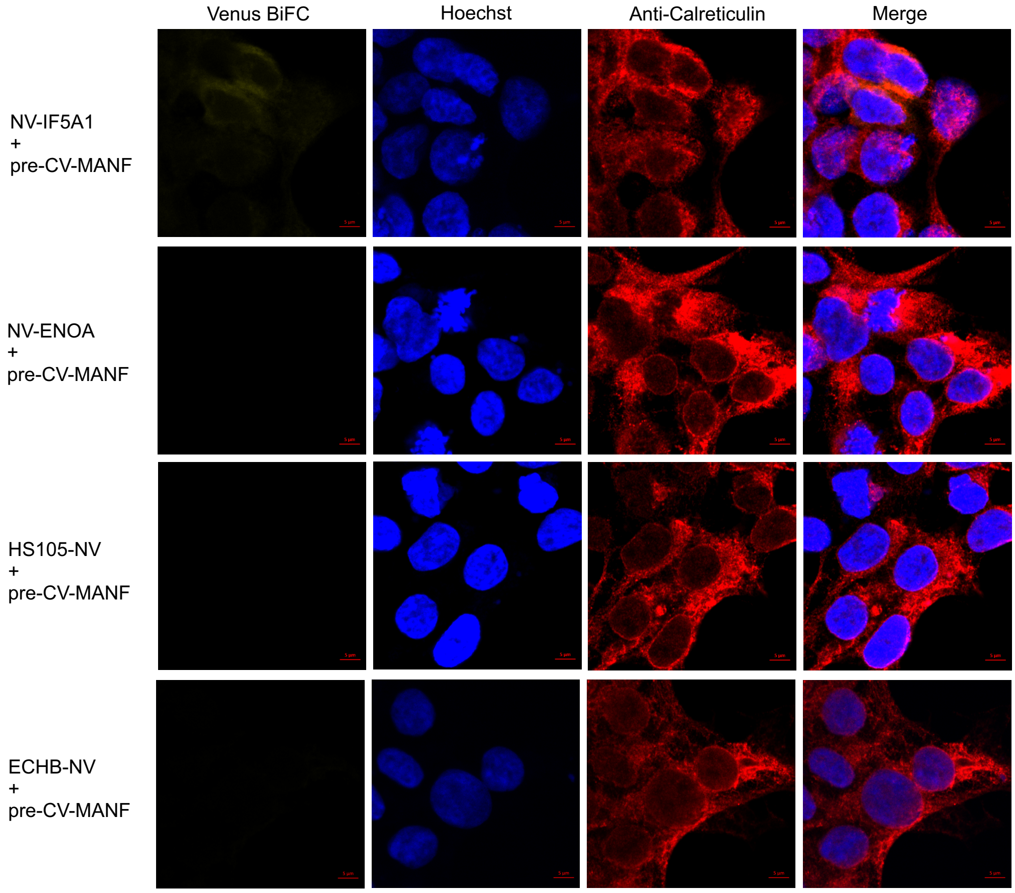

Supporting figure S2. No BiFC signal observed between MANF and proteins from the MANF conserved interactome.Red scale bars denote 10 µm.

Supporting materials and methods

Plasmids for BiFC

The following Gateway entry clones were from the Genome Biology Unit (GBU) Core Facility (Research Programs Unit, Faculty of Medicine, HiLIFE, University of Helsinki, Biocenter Finland): PGAM1 without stop (DQ896318), CKB without stop (JF432538), C1QBP without stop (DQ894514), HSPD1 without stop (DQ896771), HSPH1 with stop (DQ891727), ENO1 with stop (Dq891441), EIF5A with stop (DQ892273), HADHB without stop (DQ895971). Shown is the Genbank accession number and the presence or absence of a translation stop-codon to indicate subsequent N- or C-terminal fusion, respectively, with a Venus fragment. pDONR223-NME2 and pDONR223-PGK1 were a gift from William Hahn & David Root (Addgene plasmids # 23392 and # 23427).

MitoTracker staining

MitoTracker™ Red CMXRos (M7512, Thermo Fisher Scientific) was used according to manufacturer's instructions. Briefly, MitoTracker was added to cells in a concentration of 100 nM in warm growth media and incubated at 37^{0}C. After 15 minutes, media containing MitoTracker was removed and replaced with warm PBS. Cells were then fixed, permeabilized and mounted as described in Materials and Methods: Immunocytochemistry.
